# Molecular characterization of superficial zone chondrocytes under pro-inflammatory and biomechanical stress conditions

**DOI:** 10.1371/journal.pone.0350746

**Published:** 2026-06-12

**Authors:** Fengrui Liu, Xueting Ding, Pengcui Li, Jie Yuan, Li Guo, Sen Zhao, Gaige Wu, Xiaochun Wei

**Affiliations:** 1 Key Laboratory of Bone and Soft Tissue Injury, Second Hospital of Shanxi Medical University, Taiyuan, Shanxi, China; 2 The First Hospital of Shanxi Medical University, Taiyuan, Shanxi, China; 3 Department of Embryology, School of Basic Medical Sciences, Shanxi Medical University, Taiyuan, Shanxi, China; 4 Taiyuan Microscopic Hand Surgery Hospital, Taiyuan, Shanxi, China; The Affiliated Changzhou No 2 People’s Hospital of Nanjing Medical University, CHINA

## Abstract

Osteoarthritis (OA) is a chronic degenerative joint disease characterized by pathological features such as chondrocyte loss and cartilage matrix degradation. Superficial zone chondrocytes (SFC), located in the outermost layer of articular cartilage and in direct contact with synovial fluid, are the first to respond to mechanical stress and friction. In this study, SFC were isolated and identified in vitro, and their proliferatives and anti-apoptotic properties were examined. Additionally, an early OA inflammatory environment was successfully simulated in cell experiments, demonstrating that inflammatory conditions reduce stemness-associated marker expression in SFC, activate multiple inflammatory pathways, and promote MMP3 expression. When SFC were subjected to cyclic mechanical stretching under inflammatory conditions, increased expression of the mechanosensitive channel Piezo1, enhanced calcium-associated mechanotransduction sensitivity, and disruption of the cytoskeleton were associated with aggravated catabolic responses and apoptosis under inflammatory mechanical stimulation. These findings elucidate the role of SFC in early OA pathogenesis and provide insight into early OA pathogenesis and suggest potential directions for mechanism-based intervention.

## 1. Introduction

OA is the leading cause of disability and pain worldwide, predominantly affecting the elderly population. OA is a chronic degenerative joint disease clinically characterized by chronic pain, osteophyte formation, joint stiffness, deformity, and joint space narrowing on imaging [1,2]. The primary pathological manifestations of OA include chondrocyte loss and cartilage matrix degradation [2,3]. While multiple risk factors for OA, such as aging, obesity, osteoporosis, metabolic disorders, and sports injuries, have been explored in numerous studies [4,5], the precise pathogenesis of OA remains unclear.

Articular cartilage consists of chondrocytes embedded in an extracellular matrix composed of collagen, proteoglycans, and non-collagenous proteins [6,7]. It plays a crucial role in reducing joint friction and bearing mechanical stress. Additionally, articular cartilage maintains joint homeostasis through close material exchange with synovial fluid [8].

SFC, located in the outermost layer of articular cartilage, are the first to respond to mechanical stress and friction due to their direct contact with synovial fluid [9,10]. These cells are closely associated with OA progression and play a vital role in maintaining joint homeostasis and delaying OA development [11]. Studies have shown that SFC exhibits strong proliferative capacity, enabling self-renewal and repair of damaged cartilage following mechanical stimulation or injury [12]. Furthermore, these cells secrete proteoglycan 4 (PRG4, also known as lubricin), which not only maintains joint lubrication through its mucin-like domains but also suppresses the release of inflammatory factors by inhibiting the NF-κB signaling pathway, thereby alleviating chondrocyte inflammation [13,14]. However, further research on the anti-inflammatory and anti-apoptotic properties of SFC is still lacking.

Interleukins (IL) are a large family of cytokines, among which IL-1 is recognized as the classic pro-inflammatory cytokine, comprising 11 members, with IL-1α and IL-1β being the most extensively studied. Elevated IL-1 expression has been observed in the synovial fluid of patients with rheumatoid arthritis (RA) and OA [15,16], as well as in OA cartilage tissue [17]. During OA progression, IL-1, particularly IL-1β, plays a critical role by activating all three MAPK pathways (ERK, p38, JNK) and the NF-κB signaling cascade. It also induces other factors such as nitric oxide (NO) and prostaglandin E2 (PGE2), mediating crosstalk between IL-1β and WNT, Notch1, and TLR signaling pathways [18,19]. In vitro, IL-1β is commonly used to stimulate chondrocytes to mimic OA-like injury [20].

SFC are located at the cartilage surface and are the first to be damaged in OA, they are highly sensitive to inflammatory and biomechanical signals [21]. Inflammatory signaling affects cellular mechanosensing, making chondrocytes more susceptible to mechanical stress and impairing their adaptive responses [22]. Conversely, mechanical stress induces inflammatory reactions, leading to further chondrocyte damage. Therefore, an in vitro systematic study of SFC under inflammatory signaling and mechanotransduction holds significant implications for the early diagnosis and prevention of OA.

## 2. Methods

### 2.1. Primary cell extraction, culture and treatment

As mentioned in the previous study [23], the proximal femur and distal tibia of the knee joints of neonatal C57 mice (p5-7) were taken. Previously reported superficial chondrocyte extraction methods are summarized in S1 File. And the ligaments and tendons at the attachment sites were carefully cut with ophthalmic scissors using a body-viewing microscope, leaving the cartilaginous tissue of the proximal femur and distal tibia; The retained tissue was incubated with 0.25% trypsin for 1 h and digested to remove residual soft tissues around the epiphysis; digested with 4 mg/ml type I collagenase for 1.5 h; pre-coated dishes were inoculated with free cells (500,000 cells/100 mm dish) using a 0.1% plasma fibronectin solution for 2 h, and blocked with 3% BSA for 30 min.(α5 integrins secreted by superficial cells adhere to fibronectin) DuPont’s Modified Eagle’s Medium (DMEM) Flush unattached cells twice for 20 min each time, and incubate attached cells (i.e., superficial chondrocytes) in DMEM containing 10% fetal bovine serum. Cells from the superficial layer of cartilage tissue were passaged and cultured into uncoated petri dishes at a ratio of 1:3 to continue the culture in preparation for subsequent experiments. The remaining cartilage tissue was digested using 1.5 mg/ml I collagenase for 4 h and cultured in medium containing 10% fetal bovine serum (FBS) as a chondrocyte control (CC) for identification of SFC.

Second-passage (P2) SFC was subjected to cyclic tensile strain (CTS) intervention. Cells were seeded into silicone stretch chambers (2 × 10^5 cells/well) and treated with 20 ng/mL interleukin-1β (IL-1β) for 24 hours. The FLEXCELL-5000 mechanical stretching system was used to apply 8% or 16% CTS at 0.5 Hz for 12 hours in a CO_2_ incubator. Control cells were cultured on the same plate without IL-1β or mechanical stimulation.

All animal procedures were approved by the Ethics Committee of the Second Hospital of Shanxi Medical University (approval no. 2023YX077) and were conducted in accordance with relevant ethical guidelines and regulations.

### 2.2. Histological staining

Knee joint samples from 7-day-old C57 mice were fixed in 4% paraformaldehyde for 24 hours, dehydrated through a graded ethanol series, cleared in xylene, and embedded in paraffin. Sagittal sections (5 μm thick) were prepared using a microtome. After deparaffinization in xylene and rehydration in graded ethanol, the sections were stained as follows:

Safranin O-Fast Green Staining: Sections were stained with 0.1% Fast Green for 5 minutes and 0.2% Safranin O for 2 minutes, followed by dehydration and mounting.

Immunohistochemistry (IHC): After antigen retrieval, endogenous peroxidase blocking, and nonspecific binding blocking, sections were incubated with primary antibody against Prg4 (overnight at 4℃), followed by HRP-conjugated secondary antibody (37℃, 30 minutes). DAB was used for chromogenic detection, and nuclei were counterstained with hematoxylin. Sections were dehydrated and mounted with neutral resin.

### 2.3. Quantitative reverse transcription PCR (qRT-PCR)

Primary chondrocytes were cultured to generation 2, and 3 wells of cells were used for each group, with each well being an independent sample, and 1 mL of TRIzol™ reagent ((Invitrogen; Thermo Fisher Scientific, Inc.) was added for RNA isolation and extraction of each group of cells. The obtained RNA was then reverse transcribed into complementary DNA (cDNA) using PrimeScript™ RT Master Mix (Takara Bio, Inc.), using rRNA 18S as an internal control for mRNA. The cDNA samples were then qPCR amplified using the TB GreenTM Premix Ex TaqTM II kit(Takara) and the BiosystemsTM QuantStudioTM 6 Flex Real-Time PCR System (Applied Biosystems; Thermo Fisher Scientific, Inc.). The qPCR conditions were as follows: preincubation of samples at 95℃ for 30 s, and then 40 cycles of denaturation at 95℃ for 5 s, annealing at 60℃ for 34 s, and dissolving at 95℃ for 5 s, 65℃ for 1 min, and 97℃ for 15 s. Levels of each transcript were quantified using the threshold cycle (Ct) 2-ΔΔCq method. Primer sequences are listed in Table 1.

**Table 1:** List of primers for RT-PCR.

Gene symbol	Forward 5’-3’	Reversed 5’-3’
Mouse Prg4	CACCATCTCCACGCAGAAT	TGCTGAATGTTGCCACCTCTCTTG
Mouse ERG	GGTCTTGAAGGTCCCGATGC	CACTCTGCGCTCATTTGTGG
Mouse Tenascin C	CAGTACCACGGCTACCACAG	CATTCTCCGATGCCGTCCAG
Mouse Agg	CATGCTTATGCCTTCCGAGC	CTTTCTTCTGCCCGAGGGTT
Mouse Col9	TTGGAAGTCCGGGTGCTACT	ATCCACGAGACCCAGGTACA
Mouse Mat1	GAAGTGTGAGACCCGTGGAG	GAGCGGGAACTCTGGTTTCA
Mouse CD105	AGCTTTGTACCCACAAGTCTCG	AGACACACCTTCCAAGCGAC
Mouse CD34	GGAACCTTGATGGCTGTTGG	GTTGTCTTGCTGAATGGCCG
Mouse Sox2	AGTGGTACGTTAGGCGCTTC	CCCAGCAAGAACCCTTTCCT
Mouse Mmp3	GGAGGCAGCAGAGAACCTAC	GCAGAAGCTCCATACCAGCA
Mouse 18s	CGGCTACCACATCCAAGGAA	GCTGGAATTACCGCGGCT

### 2.4. Real-time cell analysis (RTCA)

In real-time cell analysis, a complete medium (100 µL) containing 4 × 10^3^–5 × 10^3^ cells was loaded in each well of the 96-well plate. The plate was incubated for a minimum of 30 min in a humidified (37℃) 5% CO_2_ incubator and then inserted into a real-time cell electronic sensing system; IL-1β (20ng/ml) was added to the experimental medium at 24 h, then cell survival rate was monitored to 72 h.

### 2.5. RNA-seq and bioinformatics analysis

SFC were isolated by differential adhesion and expanded to P2. The experimental group was treated with 20 ng/mL IL-1β for 16 h, while the control group remained untreated. Total RNA was extracted using TRIzol® (Magen), and RNA quality was assessed via Nanodrop ND-2000 (A260/A280 ratio) and Agilent Bioanalyzer 4150 (RIN value). Libraries were prepared using the ABclonal mRNA-seq Lib Prep Kit, fragmented, and subjected to cDNA synthesis. After PCR amplification and purification, libraries were sequenced (NovaSeq 6000, PE150). Bioinformatics analysis was performed by Shanghai Zhongke New Life Biotechnology Co.

Additionally, the GEO dataset GSE132379 was analyzed. Differentially expressed genes (DEGs) between SFC and CC groups were identified, and hierarchical clustering was performed. GO enrichment analysis (bar plots) elucidated biological functions, cellular localization, and molecular activity of DEGs. KEGG pathway analysis identified significant signaling and metabolic pathways. Gene Set Enrichment Analysis (GSEA) explored coordinated expression changes genome-wide.

### 2.6. Cell viability measurement by flow cytometry

SFC were seeded in six-well culture plates at 70%–80% confluence. Moreover, the cells were subjected to different treatments according to specific experiment requirements. These treatments include 1. DMEM/F12 containing 10% fetal FBS as control; 2.IL-1 (20 ng/mL) treated for 16h, then according to the manufacturer’s instructions, the viability of cells was detected after 48 h by PE Annexin V and 7-AAD staining kits. Stock solutions of 7-AAD and PE Annexin V were prepared. Live (orange) and dead (red) cells were detected, and the percentages of live and dead cells were quantified by a flow cytometer. The experiment was repeated thrice.

### 2.7. Western blot

Total protein was extracted from control and experimental groups, quantified by BCA assay, and 20 µg protein/lane was separated by 10% or 12.5% SDS-PAGE. Proteins were transferred to PVDF membranes (small proteins: 250 mA, 50 min; large proteins: 300 mA, 90 min). Membranes were blocked with 5% skim milk (1 h, RT), incubated with primary antibodies (overnight, 4℃) and HRP-conjugated secondary antibodies (2 h, RT), and visualized by ECL. Band intensity was analyzed using ImageJ (normalized to β-actin).

### 2.8. Statistical analysis

All experiments had ≥ 3 biological replicates. Data are presented as mean ± SD. Group comparisons used SPSS 22.0 (two-tailed t-test or one-way ANOVA with LSD post hoc test). RNA-seq DEGs were filtered at |log2FC| > 1 and FDR < 0.05. Flow cytometry data were analyzed by two-tailed t-test. GO/KEGG and GSEA used Fisher’s exact test (significance: adjusted P < 0.05). *, **, and *** denote P < 0.05, P < 0.01, and P < 0.001, respectively.

## 3. Results

### 3.1. Isolation and identification of SFC from mouse articular cartilage

**Fig 1:**
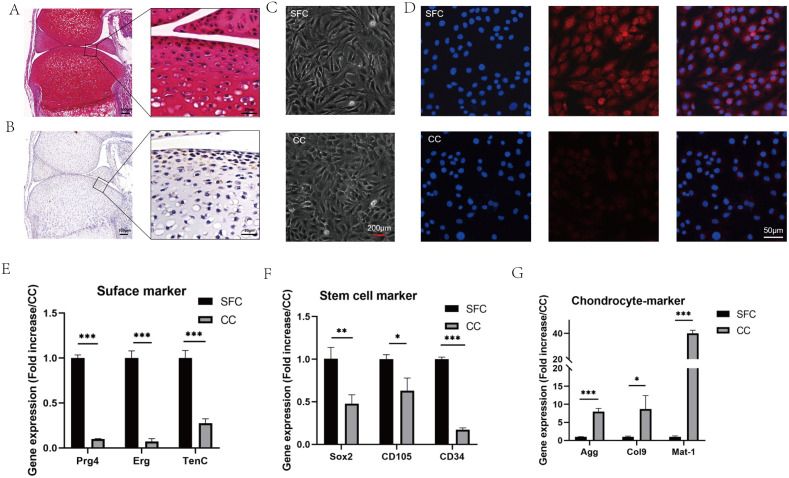
Isolation and characterization of superficial zone chondrocytes. (A) Safranin O/Fast Green staining of knee articular cartilage sections from 7-day-old C57BL/6 mice. (B) Immunohistochemical staining of neonatal mouse knee cartilage using PRG4 antibody (brown indicates positive staining, with nuclear counterstaining in blue). (C) Phase-contrast images of isolated superficial chondrocytes (SFC) and control chondrocytes (CC) after 48-hour culture via differential adhesion method. (D) Immunofluorescence staining of SFC with PRG4 antibody (green), counterstained with DAPI (blue) for nuclei. (E-G) Quantitative PCR analysis of (E) SFC markers (Prg4, Erg, Tenascin C), (F) stemness markers (CD105, CD34, Sox2), and (G) chondrogenic markers (Aggrecan, Col9a1, Matrilin-1) in SFCs versus CCs. Data presented as mean ± SD; *P < 0.05, **P < 0.01, ***P < 0.001 by Student’s t-test.

SFC with unique phenotypic characteristics were isolated and identified from mouse knee joint cartilage using differential adhesion. Safranin O-Fast Green staining revealed distinct morphological differences among chondrocytes in different layers of 7-day-old articular cartilage (Fig 1A). Immunohistochemistry (Fig 1B) and immunofluorescence (Fig 1D) confirmed that superficial zone chondrocytes exhibited high enrichment of PRG4 (a SFC-specific marker). Following 48 hours of adhesion, SFC demonstrated significant morphological differences compared to CC (Fig 1C), with SFC displaying a spindle-shaped morphology and CC exhibiting round and blunt cellular bodies, consistent with histological observations. Key gene expression profiling (qPCR, Figs 1E-G) distinctly differentiated SFCs from CCs. Relative to CCs, SFCs showed significantly elevated expression of marker genes (*Prg4, Erg, Tenascin C*) and mesenchymal stem cell markers (*CD34, CD105, Sox2*), but markedly reduced expression of crucial cartilage matrix genes (*Agg, Col9a1, Matn1*). Flow cytometry analysis further quantitatively demonstrated high PRG4 expression prevalence within the SFC population (S1 Fig).

### 3.2. Proliferative and anti-apoptotic properties of SFC

**Fig 2:**
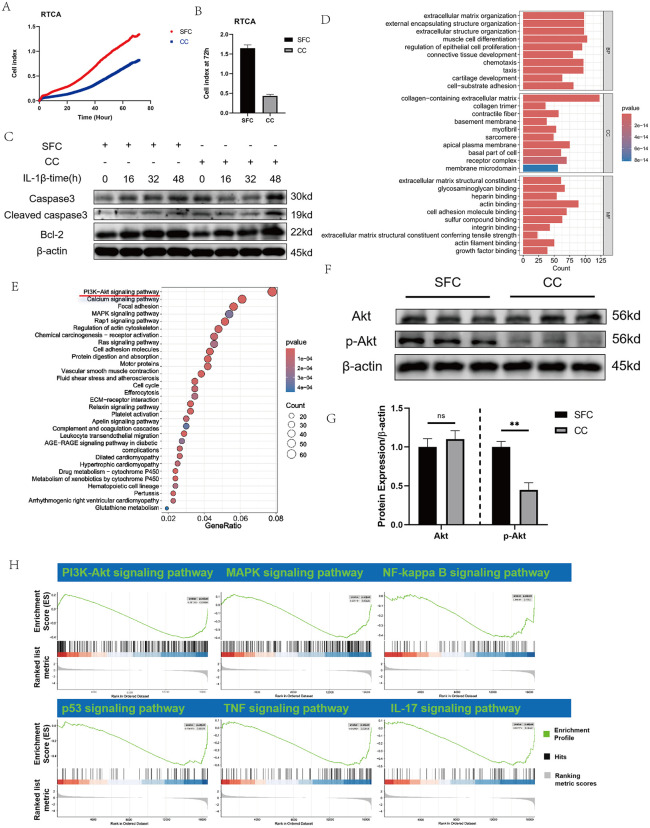
Proliferative capacity and anti-apoptotic features of SFC. (A) Real-time cell viability of SFC and CC over 72 hours measured by RTCA (Real-Time Cell Analysis). (B) Quantification of RTCA data by cell index (n = 4). (C) Western blot analysis of apoptosis markers (caspase-3/cleaved caspase-3) and anti-apoptotic marker (Bcl-2) in SFC and CC following IL-1β stimulation at different time points. (D) GO enrichment bar plot of differentially expressed genes (DEGs) between SFC and CC (data from GSE132379). (E) Functional and KEGG pathway enrichment analysis of DEGs in SFC vs. CC. (F) Western blot validation of the most significantly altered signaling pathways identified in SFC and CC. (G) Statistical analysis of Western blot band intensities from (F). (H) GSEA of DEGs between SFC and CC. Data are presented as mean ± SD; *P < 0.05, **P < 0.01, ***P < 0.001.

Compared to CC, SFC demonstrated enhanced proliferative capacity and superior resistance to inflammation-induced apoptosis. Real-time label-free cell analysis (RTCA) based on cell index revealed significantly higher cell viability in SFC versus CC during the 72-hour monitoring period (Figs 2A, B). When stimulated with IL-1β, SFC more effectively suppressed the activation of the key apoptotic protein caspase-3, reduced cleaved-caspase3 levels, and maintained higher expression of the anti-apoptotic protein Bcl-2 (Fig 2C).

In-depth bioinformatic analysis further elucidated the molecular mechanisms underlying anti-inflammatory and anti-apoptotic properties of SFC. GO enrichment analysis (Fig 2D) revealed SFC have dynamic interactions with their microenvironment, particularly highlighting adhesion and spreading processes dependent on ECM architecture. These biological processes are influenced by calcium signaling-mediated ECM remodeling and protease activity regulation, ultimately governing stem cell proliferation. KEGG pathway analysis (Fig 2E) demonstrated significant enrichment of differentially expressed genes in SFC versus CC within key signaling pathways. Notably, PI3K-AKT pathway exhibited marked activation in SFC, evidenced by elevated AKT phosphorylation (Figs 2F, G). GSEA further confirmed the activated state of PI3K-AKT signaling in SFC. Concurrently, GSEA indicated suppression of inflammatory pathways in SFC, including tumor necrosis factor alpha (TNFα) signaling, IL-17 cytokine pathway, and NF-κB signaling (Fig 2H). Additionally, the gene expression heatmap presented in S2 Fig. visually demonstrated systematic transcriptional differences between superficial zone (SFZ) and middle/deep zone (MDZ) chondrocytes.

### 3.3. Early-stage inflammatory cytokine intervention does not induce apoptosis in SFC

**Fig 3:**
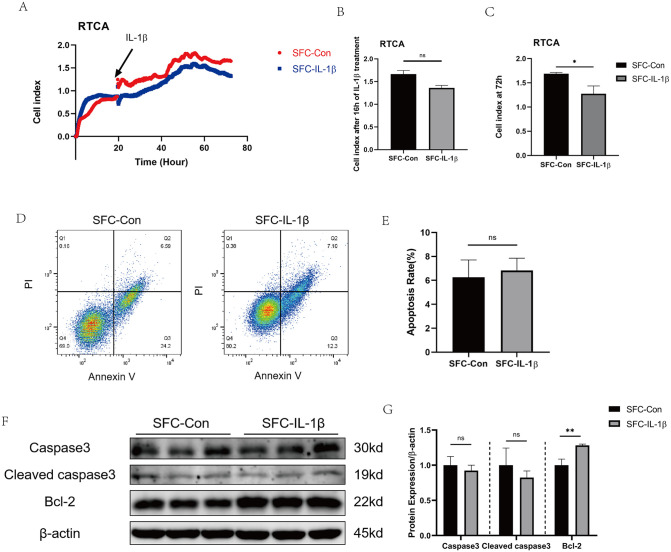
Proliferative and apoptotic characteristics of SFC under early inflammatory stimulation. (A) Real-time cell viability monitored by RTCA over 72 hours in IL-1β-treated and control groups. (B) Quantification of RTCA data by cell index at 16 hours post IL-1β treatment (n = 4). (C) Statistical analysis of 72-hour RTCA cell index data (n = 4). (D) Representative flow cytometry plots showing cell viability in control and IL-1β-treated groups at 16 hours. (E) Quantification of apoptotic cells (mean percentage, n = 3). (F) Western blot analysis of apoptosis markers (caspase-3 and cleaved caspase-3) and anti-apoptotic marker (Bcl-2). (G) Statistical analysis of Western blot band intensities from panel (F). Data are presented as mean ± SD; *P < 0.05, **P < 0.01, ***P < 0.001.

RTCA analysis revealed that IL-1β stimulation of SFC for 16 hours caused no significant difference in cell viability compared to controls (Figs 3A, B). However, extended 72-hour IL-1β exposure significantly suppressed cellular proliferation (Figs 3A, C). Flow cytometric analysis demonstrated comparable apoptotic cell proportions between IL-1β-treated and control groups at the 16-hour timepoint (Figs 3D, E). Western blotting further confirmed these observations, expression levels of pro-apoptotic proteins caspase-3 and cleaved caspase-3 showed no statistically significant changes between groups, while anti-apoptotic Bcl-2 expression was significantly elevated (Figs 3F, G). Collectively, data from cell viability assays, apoptosis quantification, and key apoptotic regulatory protein analyses demonstrate that short-term inflammatory stimulation by IL-1β does not significantly alter proliferative capacity or trigger apoptosis in superficial zone chondrocytes during the early observation window.

### 3.4. Early inflammatory intervention in SFC reduces stemness-associated marker expression, activates inflammatory pathways, and upregulates early OA biomarkers

**Fig 4:**
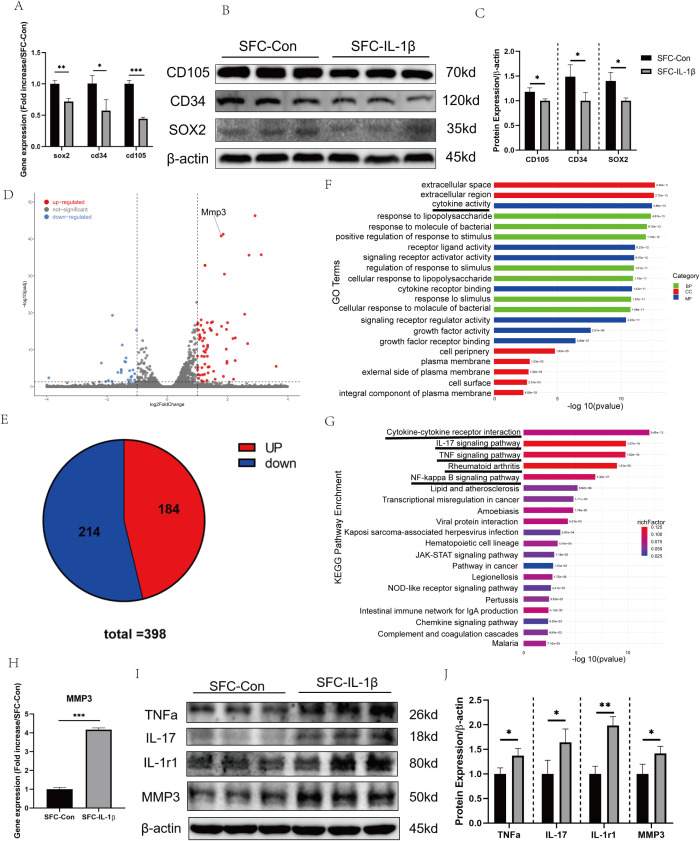
Biological characteristics of superficial chondrocytes under early inflammatory stimulation. (A) qPCR analysis of stemness-associated markers CD105, CD34, and Sox2 in control and IL-1β-treated SFC. (B) Representative Western blot images of CD105, CD34, and SOX2 protein expression, with β-actin as the loading control. (C) Densitometric analysis of CD105, CD34, and SOX2 protein levels from panel B. (D, E) RNA sequencing-based differential gene expression analysis of control and IL-1β-treated SFC. (F) GO enrichment analysis of differentially expressed genes between control and IL-1β-treated SFC. (G) KEGG pathway enrichment analysis of differentially expressed genes between control and IL-1β-treated SFC. (H) qPCR validation of the early OA biomarker MMP3. (I) Western blot analysis of inflammatory factors TNF-α and IL-17, cytokine receptor IL-1R1, and early OA marker MMP3. (J) Densitometric analysis of Western blot band intensities from panel I. Data are presented as mean ± SD; *P < 0.05, **P < 0.01, ***P < 0.001.

Early IL-1β intervention significantly reduced stemness-associated marker expression in superficial zone chondrocytes. qPCR analysis showed markedly decreased mRNA levels of CD105, CD34, and Sox2 (Fig 4A). Western blot analysis further demonstrated reduced protein expression of CD105, CD34, and SOX2 in IL-1β-treated SFC compared with controls (Fig 4B), and densitometric analysis confirmed this reduction (Fig 4C). These findings indicate that early inflammatory stimulation suppresses the progenitor-like phenotype of SFC at both the transcriptional and protein levels. Meanwhile, RNA sequencing-based differential gene expression analysis revealed activation of inflammatory pathways (Figs 4D, E). GO and KEGG enrichment analyses (Figs 4F, G) confirmed significant enrichment of inflammation-associated pathways, particularly the NF-κB signaling cascade. Experimental validation further demonstrated upregulated expression of inflammatory factors (TNF-α, IL-17) and cytokine receptor IL-1R1 (Fig 4I, statistical analyses J, and S3 Fig.). Notably, the early osteoarthritis biomarker MMP3 exhibited substantially increased expression in both qPCR (Fig 4H) and Western blot assays (Fig 4I), confirming that inflammatory stimulation promotes molecular changes associated with early osteoarthritis pathogenesis.

### 3.5. Mechanical loading under inflammatory conditions disrupts the SFC cytoskeleton and promotes apoptosis

Under pro-inflammatory conditions induced by IL-1β intervention, SFC exhibited significant pathological responses to mechanical stimulation through cyclic tensile stress (CTS). Western blot analysis (S4 Fig.) demonstrated that combined IL-1β and CTS stimulation markedly reduced the expression of anabolic marker COL2A1 while significantly increasing catabolic markers MMP3 and MMP13, as well as hypertrophy marker COL10A1, accompanied by upregulated pro-apoptotic caspase-3 and elevated anti-apoptotic Bcl-2.

**Fig 5:**
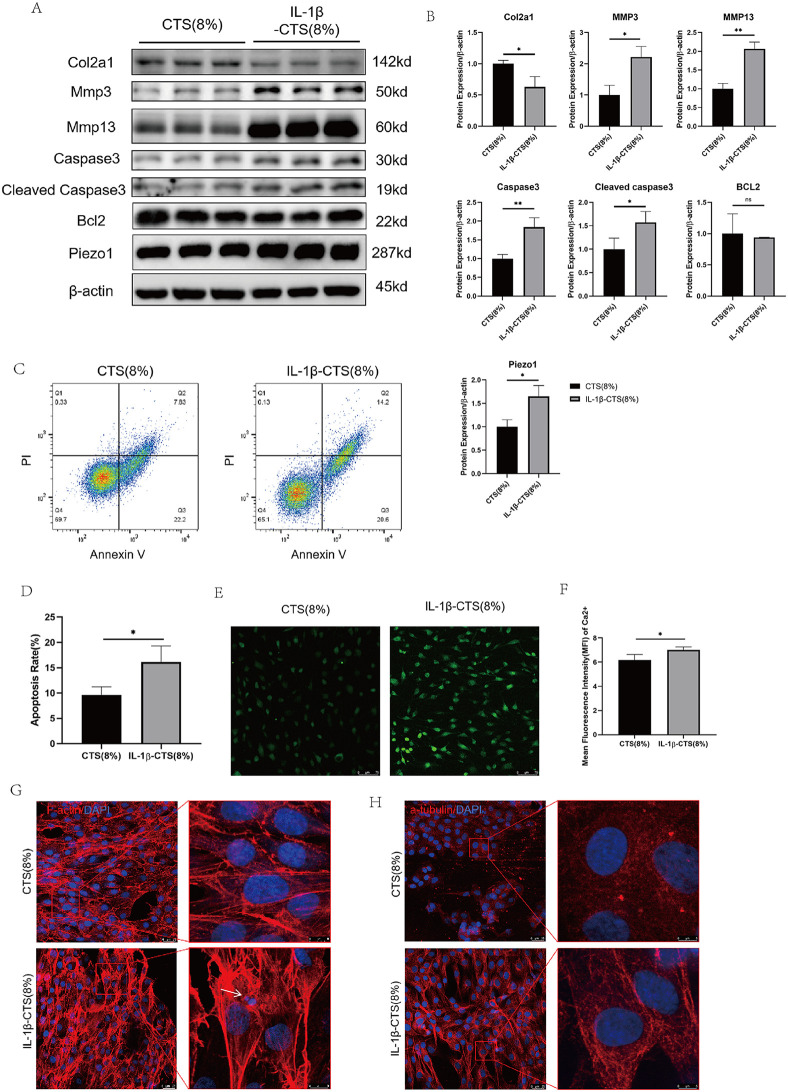
Mechanoresponsive characteristics of SFC under inflammatory conditions. (A) Total protein extracted from control and IL-1β-treated groups subjected to 8% cyclic tensile strain (CTS-8%) was analyzed by Western blot for anabolic (Col2a1), catabolic (Mmp3 & Mmp13), apoptotic (Caspase-3 & Cleaved caspase-3), anti-apoptotic (Bcl-2), and mechanosensitive ion channel (Piezo1) markers. (B) Statistical analysis of Western blot band intensities from panel (A). (C) Representative flow cytometry plots showing cell viability in control and IL-1β-treated groups after CTS-8%. (D) Quantification of apoptotic cells (mean percentage, n = 3). (E) Calcein-AM imaging of intracellular calcium levels post CTS-8% in control vs. IL-1β-treated groups. (F) Quantification of mean fluorescence intensity per cell (n = 3). (G) Rhodamine-phalloidin F-actin staining after CTS-8% (white arrows indicate nuclear fragmentation). (H) α-Tubulin-RFP tracer staining post CTS-8%. Data are presented as mean ± SD; *P < 0.05, **P < 0.01, ***P < 0.001.

Further investigation into the synergistic effects of physiological mechanical loading (CTS-8%) and IL-1β revealed more pronounced pathological changes: Western blot analysis (Fig 5A, B) showed substantially increased expression of catabolic markers MMP3 and MMP13 alongside dramatically decreased Col2α1, with elevated cleaved caspase-3 but no significant change in Bcl-2, while mechanosensitive Piezo1 channel expression was significantly upregulated. Flow cytometry (Fig 5C, D) confirmed significantly increased apoptosis rates under these conditions. Fluorescence imaging (Fig 5E, F) revealed increased calcium-associated signal intensity in the IL-1β + CTS-8% group, suggesting an enhanced calcium-related response associated with increased Piezo1 expression. Cytoskeletal staining (Fig 5G, H) demonstrated characteristic pathological restructuring, including abnormal F-actin bundling, microtubule network redistribution to the cell periphery, and nuclear fragmentation as evidenced by rhodamine-phalloidin staining. These findings collectively suggest that inflammatory priming under mechanical loading is associated with increased Piezo1 expression, enhanced Ca² ⁺ -associated fluorescence, cytoskeletal disorganization, and nuclear damage, ultimately contributing to an apoptosis-cytoskeletal disarray phenotype in SFC.

## 4. Discussion

This study systematically investigated the effects of the inflammatory factor IL-1β and mechanical stress on the biological behavior of SFC by integrating molecular biology, cellular biomechanics, and omics analysis. The key findings include: SFC exhibit a stronger resistance to apoptosis induced by inflammatory factors; IL-1β intervention in SFC at an early stage reduces stemness-associated marker expression at both the mRNA and protein levels while upregulating inflammatory factors and the early OA biomarker MMP3, thereby establishing an early catabolic priming state; and the combination of IL-1β and mechanical stimulation further aggravates ECM degradation, cytoskeletal disorganization, and apoptosis. These results suggest that the interaction between inflammatory and mechanical signals may serve as a critical regulatory node in cartilage degeneration.

SFC plays a crucial role in maintaining the integrity and function of articular cartilage while preventing its degeneration [24]. Researchers have observed that the initial changes in articular cartilage in mice models of post-traumatic arthritis occur in the superficial zone, manifesting as partial loss of SFC, swelling of remaining chondrocytes, and fibrosis of superficial tissue [25,26]. Additionally, the loss of epidermal growth factor receptor activity in SFC has been identified during early-stage OA [13,25]. This study, through RTCA and Western blot analyses, demonstrated that SFC exhibit significantly stronger anti-apoptotic capabilities compared to CC. This phenomenon suggests that SFC may resist inflammatory damage through intrinsic protective mechanisms—a finding consistent with previous studies reporting the enrichment of stem cell markers in SFC [27,28]. Activated PI3K-AKT signaling together with suppression of TNF-α/IL-17/NF-κB pathways may provide SFC with a pro-survival molecular background, partially explaining their resistance to early inflammatory apoptosis. Importantly, our work further reveals the time-dependent nature of this anti-apoptotic property, short-term IL-1β exposure does not significantly affect cell viability or apoptosis rates, whereas prolonged exposure inhibits proliferation. These findings may have implications for defining early OA intervention windows. Targeted strategies to enhance SFC survival capacity may effectively delay cartilage degeneration progression.

IL-1β significantly reduced stemness-associated marker expression while upregulating MMP3 through activation of inflammation-related signaling pathways. The early decrease in CD105, CD34, and SOX2 at both the mRNA and protein levels suggests that suppression of the progenitor-like phenotype may represent an early event in OA-related injury of SFC. Nevertheless, because progenitor function was inferred from marker expression rather than directly examined by colony-forming or lineage-differentiation assays, these findings should be interpreted as evidence of early phenotypic suppression rather than definitive loss of progenitor function. As a key effector molecule in early-stage OA, MMP3 cleaves collagen networks and may function as an early catabolic mediator that primes SFC for subsequent matrix deterioration [29]. In this context, our findings support a two-step pathogenic model: inflammatory stimulation first establishes a catabolic priming state characterized by reduced stemness-associated marker expression and increased MMP3, after which mechanical loading in the inflammatory microenvironment is associated with increased Piezo1 expression, enhanced Ca² ⁺ -associated fluorescence, cytoskeletal disorganization, and apoptosis. Thus, MMP3 may be integrated into a broader inflammation–mechanotransduction–cytoskeleton cascade rather than acting as an isolated biomarker. Although S3 Fig provided immunofluorescence validation of TNF-α, IL-17, and IL-1R1 upregulation, co-localization with MMP3 was not assessed in the present study; therefore, the spatial link between inflammatory activation and catabolic responses remains to be clarified. Furthermore, this study has established an in vitro cellular model of early OA, which will facilitate subsequent scientific investigations. Lee W. et al. investigated the response of chondrocyte Piezo1 function to mechanical stimulation in an inflammatory environment. Their study demonstrated that Piezo1 induces excessive intracellular Ca2 + influx, leading to thinning of the F-actin cytoskeleton, thereby amplifying mechanically induced microtrauma deformation [22,30]. Additionally, the research revealed that the microtubule network serves as a tension sensor—it stabilizes under tensile stress but fractures under compressive stress. These subtle structural changes reflect the cellular ability to perceive and respond to mechanical variations [31,32].

Under the inflammatory-mechanical co-stimulation conditions in this study, SFC exhibited more pronounced pathological phenotypes, characterized by suppressed ECM synthesis and enhanced catabolism. Concurrently, increased Piezo1 expression was accompanied by thickening and disorganization of F-actin stress fibers, along with abnormal peripheral aggregation of microtubule networks, which may increase the susceptibility of SFC to mechanical damage. These findings appear contradictory to the prevailing view that moderate dynamic mechanical loading protects cartilage. However, this study highlights that an inflammatory microenvironment can reverse the beneficial effects of mechanical stimulation—while physiological CTS may promote ECM homeostasis, CTS under inflammatory conditions instead accelerates catabolism and apoptosis. This suggests that clinical rehabilitation strategies should adjust mechanical intervention intensity according to inflammatory status. Furthermore, the identified association between cytoskeletal disorganization and ion channel dysfunction highlights a potential mechanistic target for future OA studies.

Several limitations of this study should be acknowledged. First, this work was based on an in vitro model and therefore may not fully recapitulate the complexity of early OA in vivo. Second, although increased Piezo1 expression was associated with enhanced calcium-associated fluorescence, cytoskeletal disorganization, and apoptosis under inflammatory-mechanical co-stimulation, pharmacological inhibition or genetic silencing of Piezo1 was not performed in this study; therefore, a definitive causal role could not be established. Third, although reductions in stemness-associated markers were examined at both the mRNA and protein levels, the progenitor-like properties of SFC were inferred from marker expression and were not directly tested using colony-forming or differentiation assays. Finally, co-localization analysis between inflammatory markers and MMP3 was not performed, and the spatial relationship between inflammatory activation and catabolic responses remains to be clarified.

## Supporting information

S1 Fig Flow cytometric identification of SFC. SFC and CC were incubated with PRG4 antibody, followed by incubation with corresponding green fluorescent secondary antibodies, and then analyzed by flow cytometry to identify SFC and CC.
(TIF)

S2 Fig Clustering analysis of differentially expressed genes in SFZ chondrocyte versus MDZ chondrocyte. (TIF)

S3 Fig Inflammatory response of SFC following early cytokine intervention. Immunofluorescence images showing inflammatory factors (TNF-α, IL-17) and cytokine receptor (IL-1r1) expression in superficial chondrocytes from control and IL-1β-treated (16-hour intervention) groups.
(TIF)

S4 Fig Response of SFC to varying mechanical loading under inflammatory conditions. Western blot analysis of SFC under different cyclic tensile strain (CTS) intensities with IL-1β treatment, showing anabolic marker Col2a1, hypertrophic chondrogenesis marker Col10a1, early inflammatory marker Mmp3, catabolic marker Mmp13, apoptotic markers Caspase-3 and Cleaved caspase-3, and anti-apoptotic marker Bcl-2.
(TIF)

S1 File Summary of superficial chondrocyte extraction methods. This file summarizes differential adhesion assay and physical cutting plus enzymatic digestion methods for superficial chondrocyte extraction.
(DOCX)
