# Supplementary figures and images for: Molecular characterization of superficial zone chondrocytes under pro-inflammatory and biomechanical stress conditions
Source: PLoS One. 2026 Jun 12;21(6):e0350746. doi: 10.1371/journal.pone.0350746 (PMC13262840; doi:10.1371/journal.pone.0350746)

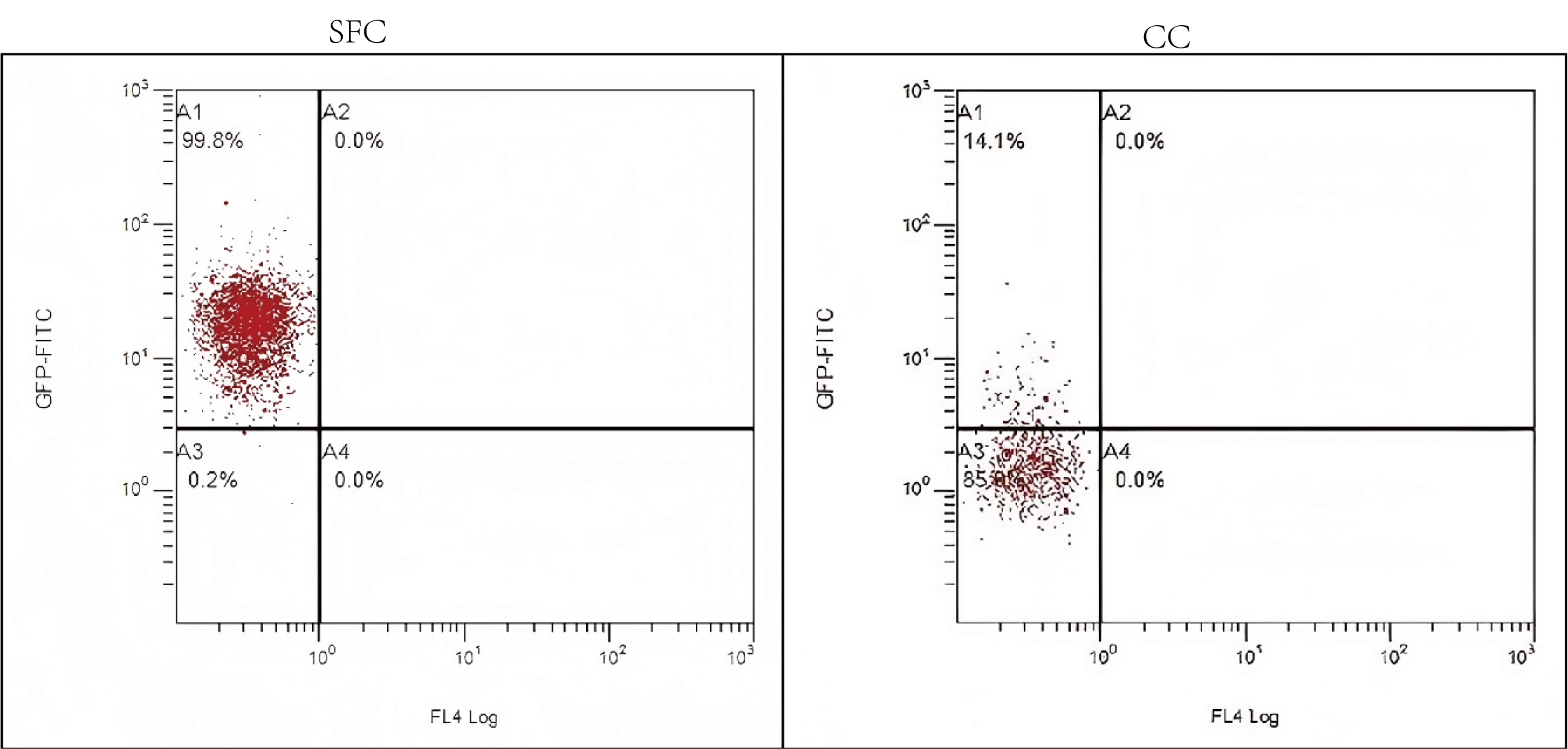

Supplement: S1 Fig — SFC and CC were incubated with PRG4 antibody, followed by incubation with corresponding green fluorescent secondary antibodies, and then analyzed by flow cytometry to identify SFC and CC. (TIF) [file pone.0350746.s001.tif]

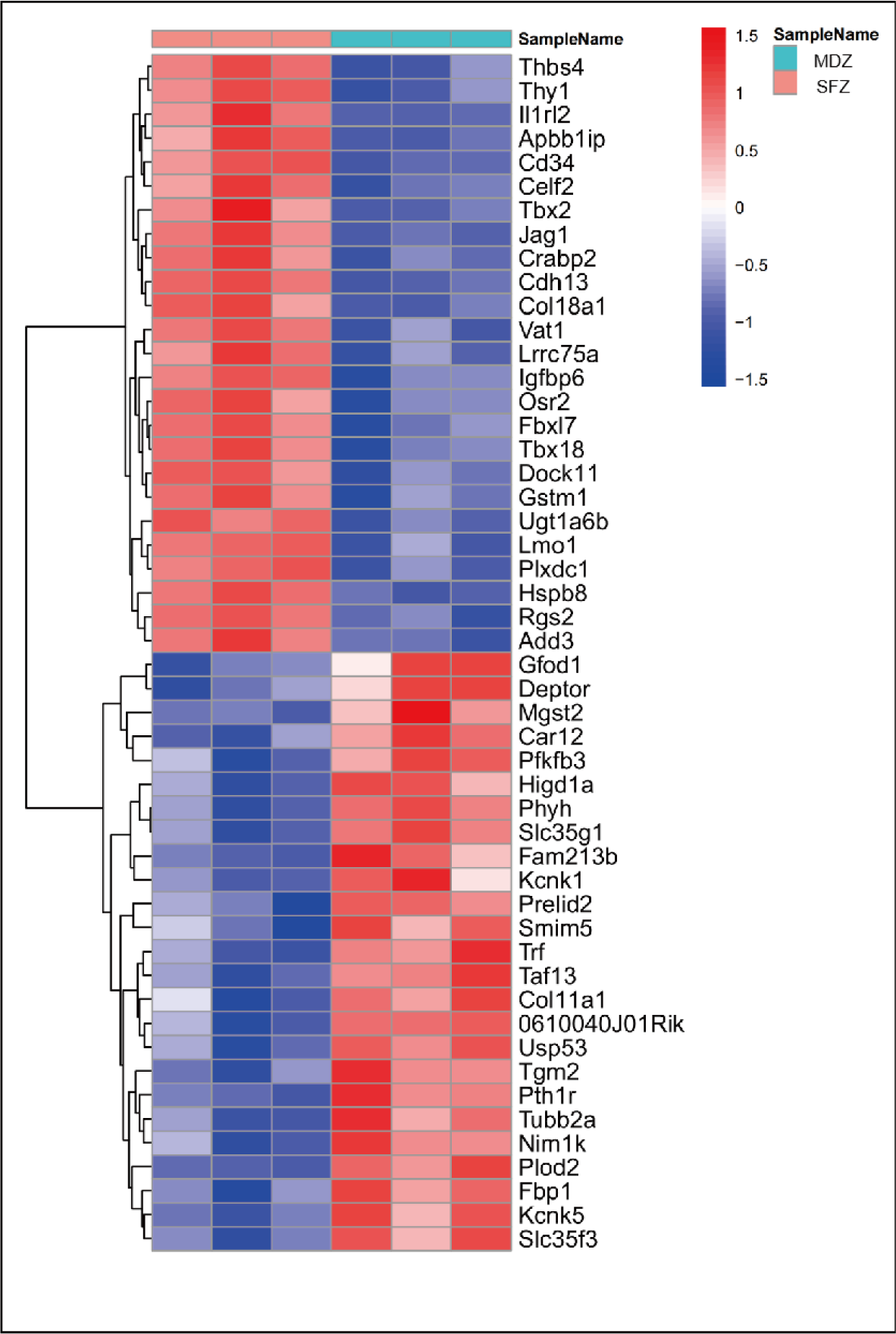

Supplement: S2 Fig — (TIF) [file pone.0350746.s002.tif]

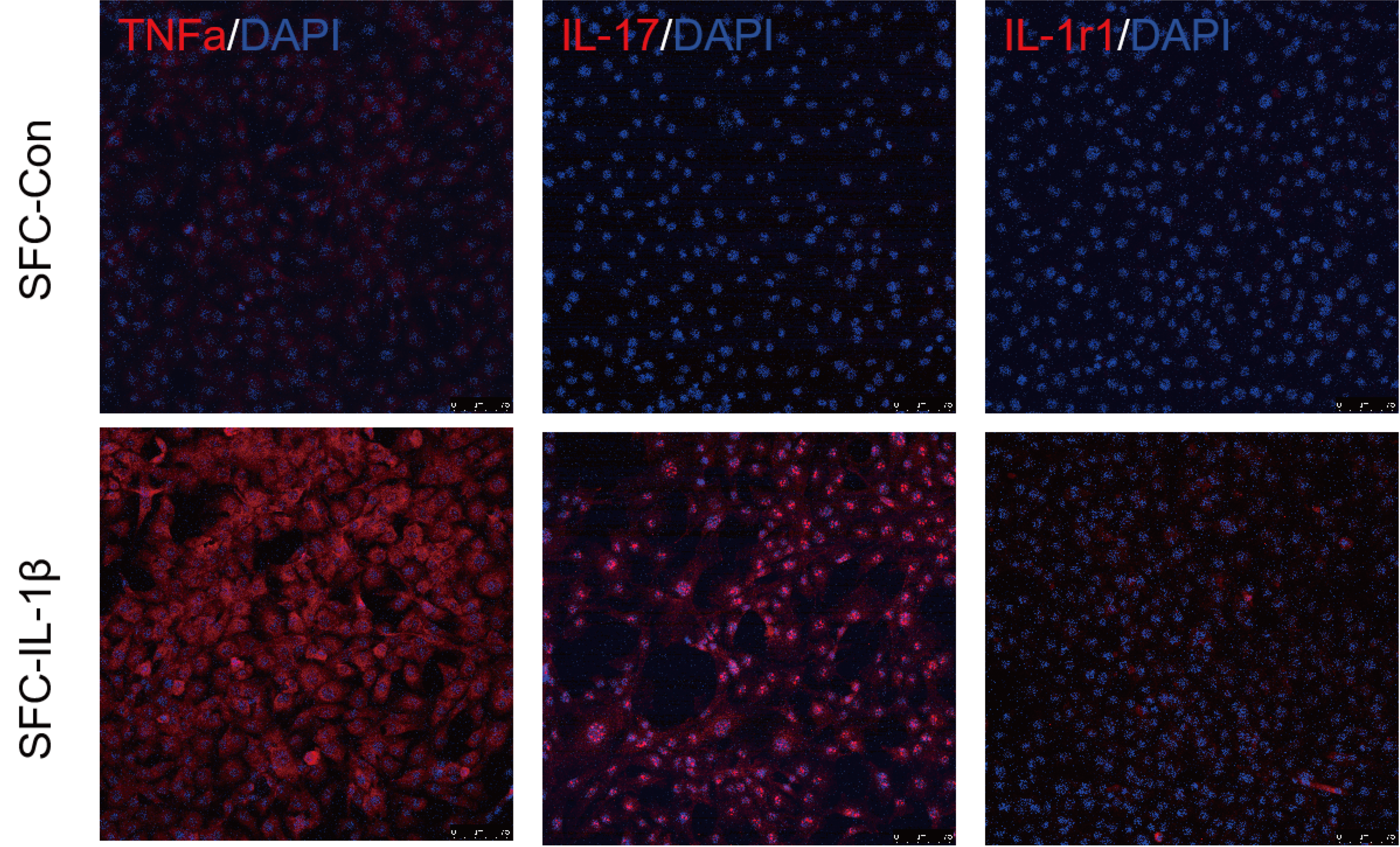

Supplement: S3 Fig — Immunofluorescence images showing inflammatory factors (TNF-α, IL-17) and cytokine receptor (IL-1r1) expression in superficial chondrocytes from control and IL-1β-treated (16-hour intervention) groups. (TIF) [file pone.0350746.s003.tif]

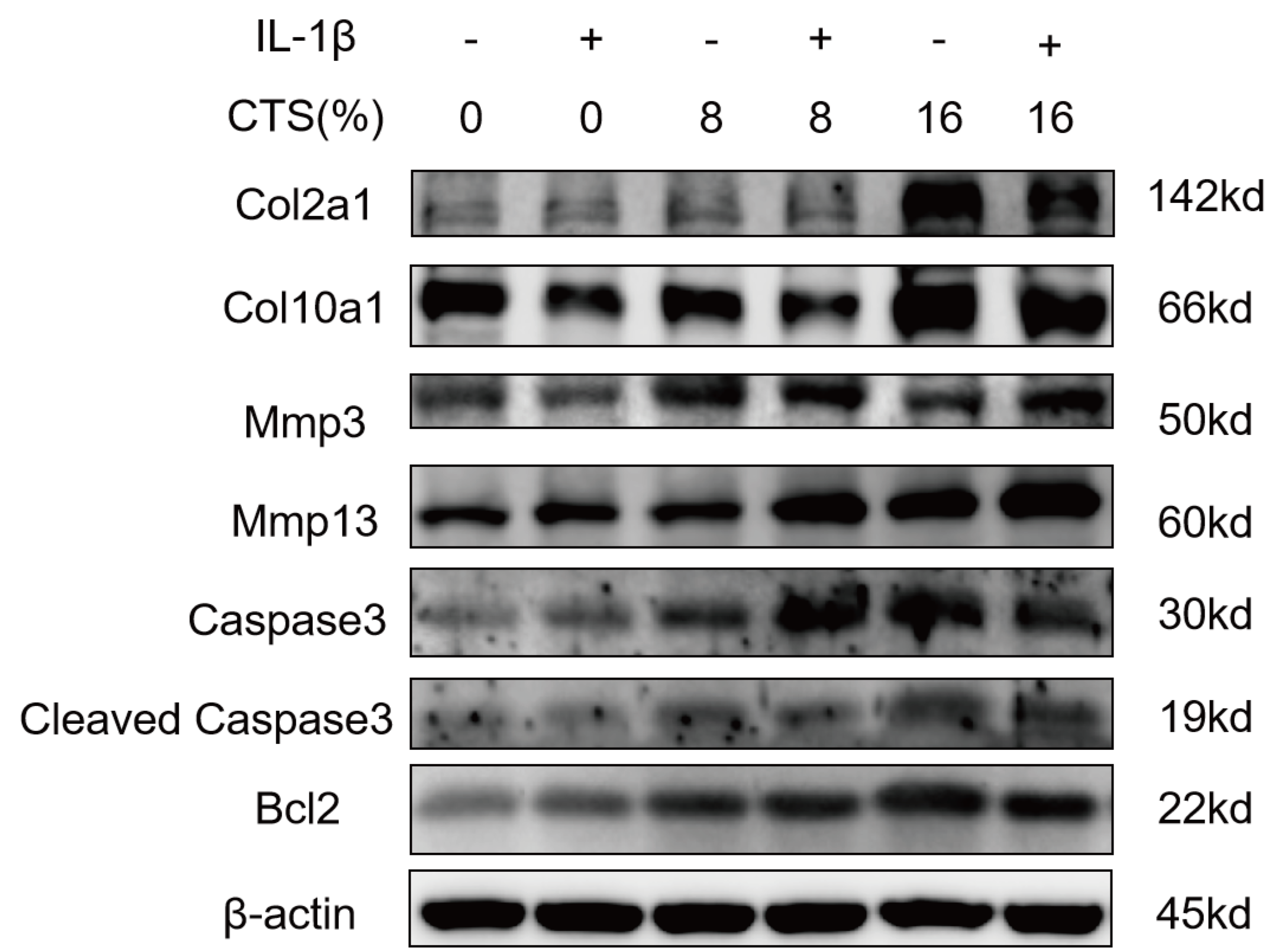

Supplement: S4 Fig — Western blot analysis of SFC under different cyclic tensile strain (CTS) intensities with IL-1β treatment, showing anabolic marker Col2a1, hypertrophic chondrogenesis marker Col10a1, early inflammatory marker Mmp3, catabolic marker Mmp13, apoptotic markers Caspase-3 and Cleaved caspase-3, and anti-apoptotic marker Bcl-2. (TIF) [file pone.0350746.s004.tif]
