# Supplementary material for: Molecular characterization of superficial zone chondrocytes under pro-inflammatory and biomechanical stress conditions
Source: PLoS One. 2026 Jun 12;21(6):e0350746. doi: 10.1371/journal.pone.0350746 (PMC13262840; doi:10.1371/journal.pone.0350746)
Supplement: S1 File — This file summarizes differential adhesion assay and physical cutting plus enzymatic digestion methods for superficial chondrocyte extraction. (DOCX) [file pone.0350746.s005.docx]

Summary of superficial chondrocyte extraction methods

**Chemical Method: Differential Adhesion Assay**

Principle: Studies have utilized the differential adhesion of cells to fibronectin in vitro to identify epidermal stem cells (Jones & Watt, 1993). In addition to the classical fibronectin receptor integrin subunits α5 and β1, fibronectin is also expressed in developing mammalian articular cartilage (Hynes, 1992). Therefore, fibronectin can be employed in vitro adhesion assays to identify and partially characterize articular cartilage progenitor cells (Archer, McDowell, Bayliss, Stephens, & Bentley, 1990).

**Table 1: Differential adhesion method for digestion of superficial cells**

| Author(s) | Journal | Subject | Purpose |
| --- | --- | --- | --- |
| (Jones & Watt, 1993) | Cell | human epidermal cell | Extraction and characterisation of epidermal progenitor cells by differential adhesion method |
| (Dowthwaite et al., 2004) | Journal of Cell Science | bovine chondrocyte surface cells | Extraction of bovine chondrocyte surface cells by differential adhesion method and characterisation of progenitor cells |
| (McCarthy, Bara, Brakspear, Singhrao, & Archer, 2012) | The Veterinary Journal | Equine chondrocyte superficial layer cells | Differential adhesion method for extracting equine chondrocytes and comparison experiments with BMSCs |
| (Yasuhara et al., 2011) | Lab Invest | Mouse cartilage superficial layer cells | Extraction of superficial cartilage cells by differential adhesion method and study of gene pathways, etc. |

**Physical Cutting + Chemical Digestion**

Principle: Using cartilage histology, cartilage tissue samples are cut in layers (with histopathological sections confirming that the collected tissues originate from different layers), followed by chemical digestive enzyme digestion of the different layers of chondrocytes, and then finally cultured and subsequent cellular experiments.

Table 2:Digested cartilage superficial layer cells after physical excision

| Author(s) | Journal | Subject | Purpose |
| --- | --- | --- | --- |
| (Archer, McDowell, Bayliss, Stephens, & Bentley, 1990) | J Cell Sci | human cartilage | The superficial and lower layers of cartilage tissue were cut and enzymatically digested to obtain cells, and it was demonstrated experimentally that the superficial cells had or partially had the ability to adhere to the difference |
| (Jiang et al., 2016) | Stem Cells Transl Med | human cartilage | Normal, OA tissues as well as articular cartilage tissues of embryonic origin were harvested in the study and screened for CSPCs by clone formation experiments using low-density implantation and passaging |

**Reference**

Archer, C. W., McDowell, J., Bayliss, M. T., Stephens, M. D., & Bentley, G. (1990). Phenotypic modulation in sub-populations of human articular chondrocytes in vitro. *J Cell Sci, 97 ( Pt 2)*, 361-371. doi:10.1242/jcs.97.2.361

Dowthwaite, G. P., Bishop, J. C., Redman, S. N., Khan, I. M., Rooney, P., Evans, D. J., . . . Archer, C. W. (2004). The surface of articular cartilage contains a progenitor cell population. *J Cell Sci, 117*(Pt 6), 889-897. doi:10.1242/jcs.00912

Hynes, R. O. (1992). Integrins: versatility, modulation, and signaling in cell adhesion. *Cell, 69*(1), 11-25. doi:10.1016/0092-8674(92)90115-s

Jiang, Y., Cai, Y., Zhang, W., Yin, Z., Hu, C., Tong, T., . . . Ouyang, H. W. (2016). Human Cartilage-Derived Progenitor Cells From Committed Chondrocytes for Efficient Cartilage Repair and Regeneration. *Stem Cells Transl Med, 5*(6), 733-744. doi:10.5966/sctm.2015-0192

Jones, P. H., & Watt, F. M. (1993). Separation of human epidermal stem cells from transit amplifying cells on the basis of differences in integrin function and expression. *Cell, 73*(4), 713-724. doi:10.1016/0092-8674(93)90251-k

McCarthy, H. E., Bara, J. J., Brakspear, K., Singhrao, S. K., & Archer, C. W. (2012). The comparison of equine articular cartilage progenitor cells and bone marrow-derived stromal cells as potential cell sources for cartilage repair in the horse. *Vet J, 192*(3), 345-351. doi:10.1016/j.tvjl.2011.08.036

Yasuhara, R., Ohta, Y., Yuasa, T., Kondo, N., Hoang, T., Addya, S., . . . Enomoto-Iwamoto, M. (2011). Roles of β-catenin signaling in phenotypic expression and proliferation of articular cartilage superficial zone cells. *Lab Invest, 91*(12), 1739-1752. doi:10.1038/labinvest.2011.144
