# Supplementary material for: Molecular characterization of superficial zone chondrocytes under pro-inflammatory and biomechanical stress conditions
Source: PLoS One. 2026 Jun 12;21(6):e0350746. doi: 10.1371/journal.pone.0350746 (PMC13262840; doi:10.1371/journal.pone.0350746)
Supplement: Attachment — Submitted filename: Revised Manuscript Submission.docx [file pone.0350746.s008.docx]

Dear Dr. Xindie Zhou and Reviewers,

Thank you for your constructive comments on our manuscript. We have carefully addressed all the concerns raised by the academic editor and reviewers. Below is our point-by-point response, with all modifications highlighted in the “Revised Manuscript with Track Changes” file.

1. Journal Requirements

1.1 Formatting Adjustments

Reformatted the manuscript using PLOS ONE templates and ensured all file names comply with journal standards.

1.2 Funding Statement

Delete funding grants that are not directly related to the article (the Shanxi Provincial Key R&D Program (202202040201012)). Updated funding information exclusively in the online submission form.

The funding source for this study was the National Natural Science Foundation of China (Grant No. U23A600019). The project leader (the corresponding author, Professor Wei) provided general oversight throughout the research, with responsibility for securing funding, supervising data management and research execution quality, and critically reviewing and revising study materials and the manuscript. As both project leader and corresponding author, Professor Wei played a pivotal supervisory and guiding role in research implementation, data stewardship, and scholarly presentation. Additional clarification can be provided upon request.

1.3 Gel/Blot Image Data

All raw images are available in Supporting Information (S1 File).

2. Reviewer’s Comments

Modify as follows:

(1) Change the title “Decreased biomechanical tolerance of superficial chondrocytes by the inflammatory factor IL-1β promotes osteoarthritis progression” to “Molecular Characterization of Superficial Zone Chondrocytes under Pro-Inflammatory and Biomechanical Stress Conditions”.

(2) Deletion of arthroscopy and staining of cartilage tissue sections derived from humans (i) to avoid ethical issues and (ii) to avoid mentioning the well-known phenomenon of superficial fibrosis in early OA.

(3) SFC was further identified by histological staining and flow cytometric identification experiments.

(4) IL-1β induces chondrocyte apoptosis, and we complemented multiple time points and control chondrocytes to highlight the early intervention of IL-1β in the SFC.

(5) We complemented cellular immunofluorescence experiments to validate the effect of IL-1β on SFC (TNFa/IL-17/IL-1r1)

(6) Superficial chondrocytes are highly resistant to apoptosis and have fluid shear stress properties in physiological state. This study systematically demonstrates the activation of ion channel Piezo1 and the reorganisation of backbone proteins when superficial chondrocytes are subjected to cyclic tension tensile stress in the early OA stage (pathological state), which is innovative and of clinically instructive significance.

(7) We improved the experimental protocol and improved the writing of the manuscript rewriting the paper.

Thank you for your understanding and support!

Best regards,

Prof. Wei
