# Supplementary material for: Molecular characterization of superficial zone chondrocytes under pro-inflammatory and biomechanical stress conditions
Source: PLoS One. 2026 Jun 12;21(6):e0350746. doi: 10.1371/journal.pone.0350746 (PMC13262840; doi:10.1371/journal.pone.0350746)
Supplement: Attachment — Submitted filename: Title Revision.docx [file pone.0350746.s007.docx]

1. Title Revision: The new title "Molecular Characterization of Superficial Zone Chondrocytes under Pro-Inflammatory and Biomechanical Stress Conditions" is more descriptive than the original. Please confirm this fully captures the study's scope, particularly the Piezo1 focus.
2. Ethical Deletion: Removing human cartilage sections was appropriate to avoid ethical concerns and superficial fibrosis discussion. Was this change also motivated by strengthening the mouse model consistency?
3. SFC Marker: PRG4 flow cytometry (Supp. Fig 1) effectively confirms SFC identity. Why was PRG4 chosen over other markers like Erg for quantitative validation?
4. Isolation Method: Differential adhesion with fibronectin coating successfully isolates SFC (spindle-shaped vs. round CC morphology). Please clarify if adhesion time (48h) was optimized or if yield/purity data could be added.
5. IL-1 Apoptosis Timing: Short-term IL-1 (16h) shows no apoptosis (RTCA, flow cytometry, caspase-3 unchanged), but 72h suppresses proliferation—this time-dependence is well-demonstrated. How does this inform early OA intervention windows?
6. Bioinformatics Pathways: GSEA/KEGG shows PI3K-AKT activation and suppressed TNF/IL-17/NF-κB in SFC vs. CC. Could the authors speculate on how this contributes to SFC's anti-apoptotic resistance?
7. Stemness Suppression: Early IL-1 markedly reduces CD105/CD34/Sox2 (qPCR Fig 4A). Does this suggest loss of progenitor potential as an early OA event, and was Sox2 protein level validated?
8. Piezo1 Mechanosensitivity: IL-1+CTS-8 upregulates Piezo1 with Ca²⁺ overload (Calcein-AM), F-actin disorganization, and apoptosis. This novel finding warrants emphasis—did inhibitors confirm Piezo1 causality?
9. Immunofluorescence Addition: New IF validation of TNF-α/IL-17/IL-1R1 upregulation (Supp. Fig 3) strengthens RNA-seq/PCR/WB data. Was co-localization with MMP3 assessed to link inflammation to catabolism?
10. MMP3 Biomarker: MMP3 upregulation by IL-1 (qPCR/WB Fig 4F/G) positions it as an early OA marker. Given its collagen-cleaving role, how does this integrate with the cytoskeleton/Piezo1 cascade in Fig 5?
