# Supplementary material for: Molecular characterization of superficial zone chondrocytes under pro-inflammatory and biomechanical stress conditions
Source: PLoS One. 2026 Jun 12;21(6):e0350746. doi: 10.1371/journal.pone.0350746 (PMC13262840; doi:10.1371/journal.pone.0350746)
Supplement: Attachment — Submitted filename: Point-by-point response.docx [file pone.0350746.s009.docx]

Our team sincerely thanks the reviewers for their detailed review and valuable suggestions regarding the manuscript. We have implemented revisions as suggested, which are detailed below:

Q1.Title Revision: The new title "Molecular Characterization of Superficial Zone Chondrocytes under Pro-Inflammatory and Biomechanical Stress Conditions" is more descriptive than the original. Please confirm this fully captures the study's scope, particularly the Piezo1 focus.

**Answer-1:** We confirm that the revised title “Molecular Characterization of Superficial Zone Chondrocytes under Pro-Inflammatory and Biomechanical Stress Conditions” comprehensively captures the study’s scope. As detailed in the manuscript, the research primarily focuses on systematically characterizing molecular responses of superficial zone chondrocytes (SFC) to simulated early OA pathological environments (pro-inflammatory cytokines and cyclic mechanical stress). This includes but is not limited to: stemness suppression, inflammatory pathway activation (e.g., NF-κB/MAPK), MMP3 upregulation, cytoskeletal disorganization, and mechanotransduction dysregulation. While Piezo1 is identified as a critical mediator of abnormal Ca²⁺ influx and mechanosensation under these conditions (Abstract: “abnormal activation of the mechanosensitive Piezo1 pathway…”; Discussion: referencing Lee et al.’s work on Piezo1 in OA pathogenesis), it represents one key component within the broader molecular landscape investigated. The title intentionally emphasizes the holistic molecular characterization objective rather than singling out individual molecules. Crucially, the core role of Piezo1 is explicitly highlighted in key sections: the Abstract directly links Piezo1 activation to catabolic responses and apoptosis; the Introduction contextualizes Piezo1 as a pivotal mechanosensor in SFC pathophysiology; and the Discussion extensively analyzes Piezo1’s contribution to the “apoptosis-cytoskeletal disarray cascade” (e.g., via Ca²⁺ overload and F-actin disruption). Thus, the title accurately reflects the study’s scope, while the manuscript text robustly establishes Piezo1’s mechanistic significance within this framework. We thank the reviewer for their insightful suggestion and confirm that the revised title is appropriate.

2.Ethical Deletion: Removing human cartilage sections was appropriate to avoid ethical concerns and superficial fibrosis discussion. Was this change also motivated by strengthening the mouse model consistency?

**Answer-2:** We confirm that deleting human cartilage data served dual purposes: ensuring ethical compliance (approved animal protocol: 2023YX077) and preserving murine model purity. This removal eliminates cross-species variables, strengthening mechanistic consistency across all experiments on mouse SFCs. We thank the reviewer for highlighting this alignment.

3.SFC Marker: PRG4 flow cytometry (Supp. Fig 1) effectively confirms SFC identity. Why was PRG4 chosen over other markers like Erg for quantitative validation?

Answer-3: We confirm PRG4’s validity for SFC identification in neonatal mice via: (1) rigorous synovial removal/pre-digestion eliminating non-SFZ contaminants; (2) stage-specific localization evidence (Kozhemyakina et al. 2015, Fig1B proving exclusive PRG4+ in SFZ at P7); (3) Alternative markers (e.g., Erg) are now contextualized as relatively enriched rather than SFZ-exclusive per literature (Trojanowska M et al. 2000; Pacifici M et al. 2006), aligning with our developmental-stage focus.

Figure 1. Model depicting the growth of articular cartilage based on Prg4GFPCreERt2 fate mapping. (Kozhemyakina E. et al. Arthritis Rheumatol. 2015)

[1] Kozhemyakina E, Zhang M, Ionescu A, Ayturk UM, Ono N, Kobayashi A, Kronenberg H, Warman ML, Lassar AB. Identification of a Prg4-expressing articular cartilage progenitor cell population in mice. Arthritis Rheumatol. 2015 May;67(5):1261-73. doi: 10.1002/art.39030.

[2] Trojanowska M. Ets factors and regulation of the extracellular matrix. Oncogene. 2000 Dec 18;19(55):6464-71. doi: 10.1038/sj.onc.1204043.

[3] Pacifici M, Koyama E, Shibukawa Y, Wu C, Tamamura Y, Enomoto-Iwamoto M, Iwamoto M. Cellular and molecular mechanisms of synovial joint and articular cartilage formation. Ann N Y Acad Sci. 2006 Apr;1068:74-86. doi: 10.1196/annals.1346.010.

4.Isolation Method: Differential adhesion with fibronectin coating successfully isolates SFC (spindle-shaped vs. round CC morphology). Please clarify if adhesion time (48h) was optimized or if yield/purity data could be added.

**Answer-4:** The adhesion period in our optimized differential adhesion method (utilizing fibronectin coating) ensured robust SFC attachment within the established principle, while streamlining workflow. This duration effectively isolated high-purity SFCs, as validated by distinct spindle-shaped morphology, positive PRG4 immunofluorescence staining and flow cytometric. Fibronectin coating enhanced adhesion efficiency, enabling specific SFC capture. Both in vitro (Fig1B) and in vivo (Fig1C & Supplementary Figure 1 ) validation by our group confirms the purity of the isolated SFC population. This method was systematically established by Dowthwaite et al. in bovine articular cartilage studies. It involves enzymatic digestion to obtain a full-thickness chondrocyte suspension, which is then seeded onto culture vessels. SFZ chondrocytes rapidly adhere within 2 hours, and non-adherent cells (primarily deep zone chondrocytes) are precisely removed within this time window, successfully yielding a high-purity population of SFZ chondrocytes. These cells highly express progenitor cell markers such as Notch1 and exhibit significant proliferation and chondrogenic potential. Subsequently, McCarthy et al. applied this technique to equine cartilage research, confirming that SFZ chondrocytes isolated via adhesion demonstrate superior expansion efficiency and chondrogenic capacity compared to BMSCs, highlighting its effectiveness in large animal models. The theoretical basis of this method can be traced back to Jones & Watt’s elucidation of the β1-integrin-mediated rapid adhesion mechanism in human epidermal progenitor cells, while Yasuhara et al. further effectively extracted mouse SFZ chondrocytes using this method.

[1] Dowthwaite GP, Bishop JC, Redman SN, Khan IM, Rooney P, Evans DJ, Haughton L, Bayram Z, Boyer S, Thomson B, Wolfe MS, Archer CW. The surface of articular cartilage contains a progenitor cell population. J Cell Sci. 2004 Feb 29;117(Pt 6):889-97. doi: 10.1242/jcs.00912.

[2] McCarthy HE, Bara JJ, Brakspear K, Singhrao SK, Archer CW. The comparison of equine articular cartilage progenitor cells and bone marrow-derived stromal cells as potential cell sources for cartilage repair in the horse. Vet J. 2012 Jun;192(3):345-51. doi: 10.1016/j.tvjl.2011.08.036.

[3] Jones PH, Watt FM. Separation of human epidermal stem cells from transit amplifying cells on the basis of differences in integrin function and expression. Cell. 1993 May 21;73(4):713-24. doi: 10.1016/0092-8674.

[4] Yasuhara R, Ohta Y, Yuasa T, Kondo N, Hoang T, Addya S, Fortina P, Pacifici M, Iwamoto M, Enomoto-Iwamoto M. Roles of β-catenin signaling in phenotypic expression and proliferation of articular cartilage superficial zone cells. Lab Invest. 2011 Dec;91(12):1739-52. doi: 10.1038/labinvest.2011.144.

5.IL-1 Apoptosis Timing: Short-term IL-1 (16h) shows no apoptosis (RTCA, flow cytometry, caspase-3 unchanged), but 72h suppresses proliferation—this time-dependence is well-demonstrated. How does this inform early OA intervention windows?

**Answer-5：**Our data suggest that IL-1β induces a staged response in SFC. At the early time point, SFC remain viable and apoptosis is not significantly increased, whereas prolonged exposure suppresses proliferative activity. Combined with the early reduction in stemness markers and upregulation of MMP3, these findings suggest that, in early OA, functional impairment of SFC may precede overt cell death. This may indicate a potentially reversible intervention window during which suppressing inflammatory signaling or preserving the progenitor-like phenotype of SFC could delay subsequent degeneration. We have revised the Discussion to clarify this point. Nevertheless, because our conclusions are derived from an in vitro model, the exact clinical intervention window still requires further in vivo validation.

6. Bioinformatics Pathways: GSEA/KEGG shows PI3K-AKT activation and suppressed TNF/IL-17/NF-κB in SFC vs. CC. Could the authors speculate on how this contributes to SFC's anti-apoptotic resistance?

**Answer-6:** We speculate that the constitutively activated PI3K-AKT pathway in SFC may provide a pro-survival background by supporting cell-survival signaling and anti-apoptotic regulators, whereas the relative suppression of TNF/IL-17/NF-κB signaling may reduce basal inflammatory stress. Together, these molecular features may help explain why SFC show higher Bcl-2 expression, lower caspase activation, and greater resistance to early inflammatory apoptosis than CC. We have added this mechanistic interpretation to the Discussion and clarified that it represents a biologically plausible interpretation based on our transcriptomic and protein data rather than direct pathway-intervention evidence.

7. Stemness Suppression: Early IL-1 markedly reduces CD105/CD34/Sox2 (qPCR Fig 4A). Does this suggest loss of progenitor potential as an early OA event, and was Sox2 protein level validated?

**Answer-7:** Thank you for this important comment. We agree that the early reduction of CD105, CD34, and SOX2 suggests that suppression of the progenitor-like phenotype may be an early event in OA-related injury of SFC. To address the reviewer’s concern, we further performed Western blot analysis of CD105, CD34, and SOX2 protein expression in control and IL-1β-treated SFC. The protein data showed reduced expression of these stemness-associated markers following early IL-1β intervention, consistent with the qPCR results (new Figure 4B, C). We have therefore revised the Results, Figure 4 legend, and Discussion accordingly. At the same time, we have intentionally used cautious wording throughout the manuscript and describe these findings as early suppression of the progenitor-like phenotype rather than definitive loss of progenitor function, because colony-forming and lineage-differentiation assays were not performed in the present study.

8. Piezo1 Mechanosensitivity: IL-1+CTS-8 upregulates Piezo1 with Ca²⁺ overload (Calcein-AM), F-actin disorganization, and apoptosis. This novel finding warrants emphasis—did inhibitors confirm Piezo1 causality?

**Answer-8:** Thank you for highlighting this important point. In the present study, we observed that IL-1β combined with CTS-8% was associated with increased Piezo1 expression, intracellular calcium overload, cytoskeletal disorganization, and apoptosis. However, we did not perform pharmacological inhibition or genetic silencing of Piezo1 in this study. Therefore, our current data support a strong association between Piezo1 upregulation and the observed pathological phenotype, but do not establish definitive causality. We have revised the Abstract, Results, and Discussion to use more cautious wording and have added this point as a study limitation. Future experiments using Piezo1 inhibitors or knockdown strategies will be necessary to confirm causality.

9. Immunofluorescence Addition: New IF validation of TNF-α/IL-17/IL-1R1 upregulation (Supp. Fig 3) strengthens RNA-seq/PCR/WB data. Was co-localization with MMP3 assessed to link inflammation to catabolism?

**Answer-9:** The purpose of Supplementary Figure 3 was to provide immunofluorescence validation of inflammatory activation indicated by RNA-seq, qPCR, and Western blot analyses. Co-localization with MMP3 was not assessed in the current study. Therefore, while our data support concurrent inflammatory activation and MMP3 upregulation following IL-1β exposure, they do not directly demonstrate intracellular co-localization between inflammatory signaling and catabolic responses. We have clarified this point in the revised Discussion and included it as a limitation.

10. MMP3 Biomarker: MMP3 upregulation by IL-1 (qPCR/WB Fig 4F/G) positions it as an early OA marker. Given its collagen-cleaving role, how does this integrate with the cytoskeleton/Piezo1 cascade in Fig 5?

**Answer-10:** Based on our findings, we propose a two-step pathogenic model. First, IL-1β exposure induces an early inflammatory-catabolic priming state in SFC, characterized by suppression of stemness-associated markers and upregulation of MMP3. Second, under this primed condition, mechanical loading further increases Piezo1 expression, calcium influx, cytoskeletal disorganization, and apoptosis, which may amplify matrix degradation and catabolic signaling. Thus, MMP3 may function as an early catabolic effector within a broader inflammation–mechanotransduction–cytoskeleton cascade. We have expanded the Discussion to better integrate MMP3 into the proposed mechanistic framework while clearly stating that this cascade remains a mechanistic interpretation rather than a fully validated linear pathway.
