# Supplementary material for: Molecular characterization of superficial zone chondrocytes under pro-inflammatory and biomechanical stress conditions
Source: PLoS One. 2026 Jun 12;21(6):e0350746. doi: 10.1371/journal.pone.0350746 (PMC13262840; doi:10.1371/journal.pone.0350746)
Supplement: Peer review history

# Peer review: Decision Letter 0

**DOI:** 10.1371/journal.pone.0350746.r001

**Authors:** Xindie Zhou

6 May 2025

PONE-D-25-07335

Decreased biomechanical tolerance of superficial chondrocytes by the inflammatory factor IL-1β promotes osteoarthritis progression

PLOS ONE

Dear Dr. Wei,

Thank you for submitting your manuscript to PLOS ONE. After careful consideration, we feel that it has merit but does not fully meet PLOS ONE’s publication criteria as it currently stands. Therefore, we invite you to submit a revised version of the manuscript that addresses the points raised during the review process.

Please submit your revised manuscript by Jun 20 2025 11:59PM. If you will need more time than this to complete your revisions, please reply to this message or contact the journal office at plosone@plos.org. When you're ready to submit your revision, log on to https://www.editorialmanager.com/pone/ and select the 'Submissions Needing Revision' folder to locate your manuscript file.

Please include the following items when submitting your revised manuscript:

- A rebuttal letter that responds to each point raised by the academic editor and reviewer(s). You should upload this letter as a separate file labeled 'Response to Reviewers'.
- A marked-up copy of your manuscript that highlights changes made to the original version. You should upload this as a separate file labeled 'Revised Manuscript with Track Changes'.
- An unmarked version of your revised paper without tracked changes. You should upload this as a separate file labeled 'Manuscript'.

If you would like to make changes to your financial disclosure, please include your updated statement in your cover letter. Guidelines for resubmitting your figure files are available below the reviewer comments at the end of this letter.

If applicable, we recommend that you deposit your laboratory protocols in protocols.io to enhance the reproducibility of your results. Protocols.io assigns your protocol its own identifier (DOI) so that it can be cited independently in the future. For instructions see: https://journals.plos.org/plosone/s/submission-guidelines#loc-laboratory-protocols. Additionally, PLOS ONE offers an option for publishing peer-reviewed Lab Protocol articles, which describe protocols hosted on protocols.io. Read more information on sharing protocols at https://plos.org/protocols?utm_medium=editorial-email&utm_source=authorletters&utm_campaign=protocols.

We look forward to receiving your revised manuscript.

Kind regards,

Xindie Zhou

Academic Editor

PLOS ONE

Journal Requirements:

When submitting your revision, we need you to address these additional requirements.

1.Please ensure that your manuscript meets PLOS ONE's style requirements, including those for file naming. The PLOS ONE style templates can be found at

https://journals.plos.org/plosone/s/file?id=wjVg/PLOSOne_formatting_sample_main_body.pdf and

https://journals.plos.org/plosone/s/file?id=ba62/PLOSOne_formatting_sample_title_authors_affiliations.pdf

2. Please note that funding information should not appear in any section or other areas of your manuscript. We will only publish funding information present in the Funding Statement section of the online submission form. Please remove any funding-related text from the manuscript.

3. Thank you for stating the following financial disclosure:

“This work was supported by the National Natural Science Foundation of China (U23A600019) and the Shanxi Provincial Key R&D Program (202202040201012).”

Please state what role the funders took in the study.  If the funders had no role, please state: "The funders had no role in study design, data collection and analysis, decision to publish, or preparation of the manuscript."

If this statement is not correct you must amend it as needed.

Please include this amended Role of Funder statement in your cover letter; we will change the online submission form on your behalf.

4. When completing the data availability statement of the submission form, you indicated that you will make your data available on acceptance. We strongly recommend all authors decide on a data sharing plan before acceptance, as the process can be lengthy and hold up publication timelines. Please note that, though access restrictions are acceptable now, your entire data will need to be made freely accessible if your manuscript is accepted for publication. This policy applies to all data except where public deposition would breach compliance with the protocol approved by your research ethics board. If you are unable to adhere to our open data policy, please kindly revise your statement to explain your reasoning and we will seek the editor's input on an exemption. Please be assured that, once you have provided your new statement, the assessment of your exemption will not hold up the peer review process.

5. PLOS ONE now requires that authors provide the original uncropped and unadjusted images underlying all blot or gel results reported in a submission’s figures or Supporting Information files. This policy and the journal’s other requirements for blot/gel reporting and figure preparation are described in detail at https://journals.plos.org/plosone/s/figures#loc-blot-and-gel-reporting-requirements and https://journals.plos.org/plosone/s/figures#loc-preparing-figures-from-image-files. When you submit your revised manuscript, please ensure that your figures adhere fully to these guidelines and provide the original underlying images for all blot or gel data reported in your submission. See the following link for instructions on providing the original image data: https://journals.plos.org/plosone/s/figures#loc-original-images-for-blots-and-gels.

In your cover letter, please note whether your blot/gel image data are in Supporting Information or posted at a public data repository, provide the repository URL if relevant, and provide specific details as to which raw blot/gel images, if any, are not available. Email us at plosone@plos.org if you have any questions.

[Note: HTML markup is below. Please do not edit.]

Reviewers' comments:

Reviewer's Responses to Questions

**Comments to the Author**

1. Is the manuscript technically sound, and do the data support the conclusions?

The manuscript must describe a technically sound piece of scientific research with data that supports the conclusions. Experiments must have been conducted rigorously, with appropriate controls, replication, and sample sizes. The conclusions must be drawn appropriately based on the data presented.

Reviewer #1: Partly

**********

2. Has the statistical analysis been performed appropriately and rigorously?

Reviewer #1: I Don't Know

**********

3. Have the authors made all data underlying the findings in their manuscript fully available?

The PLOS Data policy requires authors to make all data underlying the findings described in their manuscript fully available without restriction, with rare exception (please refer to the Data Availability Statement in the manuscript PDF file). The data should be provided as part of the manuscript or its supporting information, or deposited to a public repository. For example, in addition to summary statistics, the data points behind means, medians and variance measures should be available. If there are restrictions on publicly sharing data—e.g. participant privacy or use of data from a third party—those must be specified.

Reviewer #1: Yes

**********

4. Is the manuscript presented in an intelligible fashion and written in standard English?

PLOS ONE does not copyedit accepted manuscripts, so the language in submitted articles must be clear, correct, and unambiguous. Any typographical or grammatical errors should be corrected at revision, so please note any specific errors here.

Reviewer #1: No

**********

5. Review Comments to the Author

Please use the space provided to explain your answers to the questions above. You may also include additional comments for the author, including concerns about dual publication, research ethics, or publication ethics. (Please upload your review as an attachment if it exceeds 20,000 characters)

Reviewer #1: In this manuscript, the authors tried to investigate the response of superfacial chondrocytes of the mouse knee joint to cyclic compressive stress in the presence of the inflammatory cytokine IL-1beta. The title “Decreased biomechanical tolerance of superficial chondrocytes by the inflammatory factor IL-1β promotes osteoarthritis progression” is inaccurate, as the current results can not support the claim.

Fig. 1: The authors should justify how they obtained normal human articular cartilage. Were the normal and early OA cartilage from the same or different hosts? Also, the fibrosis of early OA cartilage is well-known in OA.

Fig. 2: If the authors want to study superficial chondrocytes, they should be purified by cell cytometry or other methods.

Fig. 3: IL-1beta is well-known to induce the apoptosis of chondrocytes. Additional time-points should be tested in 3C-F.

Fig. 4: The effects of IL-1beta on gene expression of chondrocytes have been widely studied. The authors should clarify the novelty of these results. In addition, the levels of TNFa, IL6, IL1r1, and MMP3 should be shown by ELISA or a similar technique.

Fig. 5: The influence of IL-1beta on the expression of Piezo1 and the mechanical stress on F-actin in chondrocytes should be reported in other studies. The authors should state the novelty of their results. Otherwise, the comparison of superficial chondrocytes and chondrocytes in different layers of the articular cartilage should be performed.

In addition, the writing of the manuscript should be improved.

**********

6. PLOS authors have the option to publish the peer review history of their article (what does this mean?). If published, this will include your full peer review and any attached files.

If you choose “no”, your identity will remain anonymous but your review may still be made public.

**Do you want your identity to be public for this peer review?**  For information about this choice, including consent withdrawal, please see our Privacy Policy.

Reviewer #1: No

**********

[NOTE: If reviewer comments were submitted as an attachment file, they will be attached to this email and accessible via the submission site. Please log into your account, locate the manuscript record, and check for the action link "View Attachments". If this link does not appear, there are no attachment files.]

While revising your submission, please upload your figure files to the Preflight Analysis and Conversion Engine (PACE) digital diagnostic tool, https://pacev2.apexcovantage.com/. PACE helps ensure that figures meet PLOS requirements. To use PACE, you must first register as a user. Registration is free. Then, login and navigate to the UPLOAD tab, where you will find detailed instructions on how to use the tool. If you encounter any issues or have any questions when using PACE, please email PLOS at figures@plos.org. Please note that Supporting Information files do not need this step.

# Author comment: Author response to Decision Letter 1

**DOI:** 10.1371/journal.pone.0350746.r002

27 Oct 2025

1. Journal Requirements

1.1 Formatting Adjustments

Reformatted the manuscript using PLOS ONE templates and ensured all file names comply with journal standards.

1.2 Funding Statement

Delete funding grants that are not directly related to the article (the Shanxi Provincial Key R&D Program (202202040201012)). Updated funding information exclusively in the online submission form.

1.3 Gel/Blot Image Data

All raw images are available in Supporting Information (S1 File).

2. Reviewer’s Comments

Modify as follows:

(1) Change the title “Decreased biomechanical tolerance of superficial chondrocytes by the inflammatory factor IL-1β promotes osteoarthritis progression” to “Molecular Characterization of Superficial Zone Chondrocytes under Pro-Inflammatory and Biomechanical Stress Conditions”.

(2) Deletion of arthroscopy and staining of cartilage tissue sections derived from humans (i) to avoid ethical issues and (ii) to avoid mentioning the well-known phenomenon of superficial fibrosis in early OA.

(3) SFC was further identified by histological staining and flow cytometric identification experiments.

(4) IL-1β induces chondrocyte apoptosis, and we complemented multiple time points and control chondrocytes to highlight the early intervention of IL-1β in the SFC.

(5) We complemented cellular immunofluorescence experiments to validate the effect of IL-1β on SFC (TNFa/IL-17/IL-1r1)

(6) Superficial chondrocytes are highly resistant to apoptosis and have fluid shear stress properties in physiological state. This study systematically demonstrates the activation of ion channel Piezo1 and the reorganisation of backbone proteins when superficial chondrocytes are subjected to cyclic tension tensile stress in the early OA stage (pathological state), which is innovative and of clinically instructive significance.

(7) We improved the experimental protocol and improved the writing of the manuscript rewriting the paper.

Attachment Submitted filename: Revised Manuscript Submission.docx

# Peer review: Decision Letter 1

**DOI:** 10.1371/journal.pone.0350746.r003

**Authors:** Xindie Zhou

17 Mar 2026

PONE-D-25-07335R1

Molecular Characterization of Superficial Zone Chondrocytes under Pro-Inflammatory and Biomechanical Stress Conditions

PLOS One

Dear Dr. Wei,

Thank you for submitting your manuscript to PLOS ONE. After careful consideration, we feel that it has merit but does not fully meet PLOS ONE’s publication criteria as it currently stands. Therefore, we invite you to submit a revised version of the manuscript that addresses the points raised during the review process.

==============================

This manuscript contains a change in the author's order. When revising the manuscript, please provide an explanation for this action and clarify the reason for your authorship modification.

Please ensure that your decision is justified on PLOS ONE’s publication criteria and not, for example, on novelty or perceived impact.

For Lab, Study and Registered Report Protocols: These article types are not expected to include results but may include pilot data.

==============================

Please submit your revised manuscript by May 01 2026 11:59PM. If you will need more time than this to complete your revisions, please reply to this message or contact the journal office at plosone@plos.org. When you're ready to submit your revision, log on to https://www.editorialmanager.com/pone/ and select the 'Submissions Needing Revision' folder to locate your manuscript file.

Please include the following items when submitting your revised manuscript:

- A letter that responds to each point raised by the academic editor and reviewer(s). You should upload this letter as a separate file labeled 'Response to Reviewers'.
- A marked-up copy of your manuscript that highlights changes made to the original version. You should upload this as a separate file labeled 'Revised Manuscript with Track Changes'.
- An unmarked version of your revised paper without tracked changes. You should upload this as a separate file labeled 'Manuscript'.

If you would like to make changes to your financial disclosure, please include your updated statement in your cover letter. Guidelines for resubmitting your figure files are available below the reviewer comments at the end of this letter.

If applicable, we recommend that you deposit your laboratory protocols in protocols.io to enhance the reproducibility of your results. Protocols.io assigns your protocol its own identifier (DOI) so that it can be cited independently in the future. For instructions see: https://journals.plos.org/plosone/s/submission-guidelines#loc-laboratory-protocols. Additionally, PLOS ONE offers an option for publishing peer-reviewed Lab Protocol articles, which describe protocols hosted on protocols.io. Read more information on sharing protocols at https://plos.org/protocols?utm_medium=editorial-email&utm_source=authorletters&utm_campaign=protocols.

We look forward to receiving your revised manuscript.

Kind regards,

Xindie Zhou

Academic Editor

PLOS One

Journal Requirements:

If the reviewer comments include a recommendation to cite specific previously published works, please review and evaluate these publications to determine whether they are relevant and should be cited. There is no requirement to cite these works unless the editor has indicated otherwise.

[Note: HTML markup is below. Please do not edit.]

Reviewers' comments:

Reviewer's Responses to Questions

**Comments to the Author**

1. If the authors have adequately addressed your comments raised in a previous round of review and you feel that this manuscript is now acceptable for publication, you may indicate that here to bypass the “Comments to the Author” section, enter your conflict of interest statement in the “Confidential to Editor” section, and submit your "Accept" recommendation.

Reviewer #2: All comments have been addressed

**********

2. Is the manuscript technically sound, and do the data support the conclusions?

The manuscript must describe a technically sound piece of scientific research with data that supports the conclusions. Experiments must have been conducted rigorously, with appropriate controls, replication, and sample sizes. The conclusions must be drawn appropriately based on the data presented.

Reviewer #2: Yes

**********

3. Has the statistical analysis been performed appropriately and rigorously?

Reviewer #2: Yes

**********

4. Have the authors made all data underlying the findings in their manuscript fully available?

The PLOS Data policy requires authors to make all data underlying the findings described in their manuscript fully available without restriction, with rare exception (please refer to the Data Availability Statement in the manuscript PDF file). The data should be provided as part of the manuscript or its supporting information, or deposited to a public repository. For example, in addition to summary statistics, the data points behind means, medians and variance measures should be available. If there are restrictions on publicly sharing data—e.g. participant privacy or use of data from a third party—those must be specified.

Reviewer #2: Yes

**********

5. Is the manuscript presented in an intelligible fashion and written in standard English?

PLOS ONE does not copyedit accepted manuscripts, so the language in submitted articles must be clear, correct, and unambiguous. Any typographical or grammatical errors should be corrected at revision, so please note any specific errors here.

Reviewer #2: Yes

**********

6. Review Comments to the Author

Please use the space provided to explain your answers to the questions above. You may also include additional comments for the author, including concerns about dual publication, research ethics, or publication ethics. (Please upload your review as an attachment if it exceeds 20,000 characters)

Reviewer #2: This study systematically investigates the molecular mechanisms of superficial zone chondrocytes (SFCs) under the combined effects of inflammation and mechanical stimulation. The topic closely addresses the core issue of the early pathogenesis of osteoarthritis (OA), with clear scientific significance and potential clinical translational value. Overall, the article has a rigorous structure, comprehensive experimental design, solid data, and clear logic, making it a high-quality research paper.

Advantages

1.The author focuses the research on the superficial zone chondrocytes (SFC) at the outermost layer of articular cartilage, which are the first to be exposed to inflammatory and mechanical stress environments, rather than the traditional overall chondrocyte population. This layered perspective helps deepen the understanding of the early pathological initiation mechanisms of OA and is highly cutting-edge.

2. Not only were the effects of IL-1β inflammatory stimulation and mechanical stretching (CTS) on SFC examined separately, but more importantly, a model of combined inflammatory and mechanical action was established, revealing the synergistic amplifying effect between the two. This makes the study closer to the actual pathological environment and enhances its physiological relevance.

3. This study successfully isolated superficial zone chondrocytes (SFCs) using differential adhesion and systematically verified their cellular characteristics on multiple levels. The authors not only confirmed their molecular identity through the expression analysis of superficial zone-specific markers such as PRG4, but also conducted multidimensional characterization by combining cell morphological features, stemness-related markers, and the expression profile of cartilage matrix-related genes, thereby providing substantial evidence that the obtained cell population possesses clear and stable superficial zone characteristics. This layered identification strategy enhances the reliability of the cell source and lays a solid foundation for subsequent mechanistic studies.

Meanwhile, the authors did not stop at phenotypic confirmation, but further systematically evaluated the functional changes of SFCs under inflammatory and mechanical stimuli using various techniques.

Areas for improvement

1. The article uses CTS but does not explain adequately whether it simulates physiological load or pathological mechanical stress.

2. It is recommended to further clarify the specific number of biological replicates (n values) for each key experiment in the Methods section, especially for RNA-seq and Western blot, to enhance data transparency and reproducibility.

3. It is recommended to standardize certain terms; for example, 'IL-1' and 'IL-1β' should be used consistently throughout. Some sentences are slightly verbose and could be appropriately simplified.

The above issues are all related to technical details or improvements in expression and do not affect the overall conclusions or scientific value of the article. After minor revisions, the article will be more rigorous, clear, and persuasive.

**********

7. PLOS authors have the option to publish the peer review history of their article (what does this mean?). If published, this will include your full peer review and any attached files.

If you choose “no”, your identity will remain anonymous but your review may still be made public.

**Do you want your identity to be public for this peer review?**  For information about this choice, including consent withdrawal, please see our Privacy Policy.

Reviewer #2: No

**********

[NOTE: If reviewer comments were submitted as an attachment file, they will be attached to this email and accessible via the submission site. Please log into your account, locate the manuscript record, and check for the action link "View Attachments". If this link does not appear, there are no attachment files.]

To ensure your figures meet our technical requirements, please review our figure guidelines: https://journals.plos.org/plosone/s/figures

You may also use PLOS’s free figure tool, NAAS, to help you prepare publication quality figures: https://journals.plos.org/plosone/s/figures#loc-tools-for-figure-preparation.

NAAS will assess whether your figures meet our technical requirements by comparing each figure against our figure specifications.

Attachment Submitted filename: Title Revision.docx

# Author comment: Author response to Decision Letter 2

**DOI:** 10.1371/journal.pone.0350746.r004

9 May 2026

Our team sincerely thanks the reviewers for their detailed review and valuable suggestions regarding the manuscript. We have implemented revisions as suggested, which are detailed below:

Q1.Title Revision: The new title "Molecular Characterization of Superficial Zone Chondrocytes under Pro-Inflammatory and Biomechanical Stress Conditions" is more descriptive than the original. Please confirm this fully captures the study's scope, particularly the Piezo1 focus.

Answer-1: We confirm that the revised title “Molecular Characterization of Superficial Zone Chondrocytes under Pro-Inflammatory and Biomechanical Stress Conditions” comprehensively captures the study’s scope. As detailed in the manuscript, the research primarily focuses on systematically characterizing molecular responses of superficial zone chondrocytes (SFC) to simulated early OA pathological environments (pro-inflammatory cytokines and cyclic mechanical stress). This includes but is not limited to: stemness suppression, inflammatory pathway activation (e.g., NF-κB/MAPK), MMP3 upregulation, cytoskeletal disorganization, and mechanotransduction dysregulation. While Piezo1 is identified as a critical mediator of abnormal Ca²⁺ influx and mechanosensation under these conditions (Abstract: “abnormal activation of the mechanosensitive Piezo1 pathway…”; Discussion: referencing Lee et al.’s work on Piezo1 in OA pathogenesis), it represents one key component within the broader molecular landscape investigated. The title intentionally emphasizes the holistic molecular characterization objective rather than singling out individual molecules. Crucially, the core role of Piezo1 is explicitly highlighted in key sections: the Abstract directly links Piezo1 activation to catabolic responses and apoptosis; the Introduction contextualizes Piezo1 as a pivotal mechanosensor in SFC pathophysiology; and the Discussion extensively analyzes Piezo1’s contribution to the “apoptosis-cytoskeletal disarray cascade” (e.g., via Ca²⁺ overload and F-actin disruption). Thus, the title accurately reflects the study’s scope, while the manuscript text robustly establishes Piezo1’s mechanistic significance within this framework. We thank the reviewer for their insightful suggestion and confirm that the revised title is appropriate.

2.Ethical Deletion: Removing human cartilage sections was appropriate to avoid ethical concerns and superficial fibrosis discussion. Was this change also motivated by strengthening the mouse model consistency?

Answer-2: We confirm that deleting human cartilage data served dual purposes: ensuring ethical compliance (approved animal protocol: 2023YX077) and preserving murine model purity. This removal eliminates cross-species variables, strengthening mechanistic consistency across all experiments on mouse SFCs. We thank the reviewer for highlighting this alignment.

3.SFC Marker: PRG4 flow cytometry (Supp. Fig 1) effectively confirms SFC identity. Why was PRG4 chosen over other markers like Erg for quantitative validation?

Answer-3: We confirm PRG4’s validity for SFC identification in neonatal mice via: (1) rigorous synovial removal/pre-digestion eliminating non-SFZ contaminants; (2) stage-specific localization evidence (Kozhemyakina et al. 2015, Fig1B proving exclusive PRG4+ in SFZ at P7); (3) Alternative markers (e.g., Erg) are now contextualized as relatively enriched rather than SFZ-exclusive per literature (Trojanowska M et al. 2000; Pacifici M et al. 2006), aligning with our developmental-stage focus.

Figure 1. Model depicting the growth of articular cartilage based on Prg4GFPCreERt2 fate mapping. (Kozhemyakina E. et al. Arthritis Rheumatol. 2015)

[1] Kozhemyakina E, Zhang M, Ionescu A, Ayturk UM, Ono N, Kobayashi A, Kronenberg H, Warman ML, Lassar AB. Identification of a Prg4-expressing articular cartilage progenitor cell population in mice. Arthritis Rheumatol. 2015 May;67(5):1261-73. doi: 10.1002/art.39030.

[2] Trojanowska M. Ets factors and regulation of the extracellular matrix. Oncogene. 2000 Dec 18;19(55):6464-71. doi: 10.1038/sj.onc.1204043.

[3] Pacifici M, Koyama E, Shibukawa Y, Wu C, Tamamura Y, Enomoto-Iwamoto M, Iwamoto M. Cellular and molecular mechanisms of synovial joint and articular cartilage formation. Ann N Y Acad Sci. 2006 Apr;1068:74-86. doi: 10.1196/annals.1346.010.

4.Isolation Method: Differential adhesion with fibronectin coating successfully isolates SFC (spindle-shaped vs. round CC morphology). Please clarify if adhesion time (48h) was optimized or if yield/purity data could be added.

Answer-4: The adhesion period in our optimized differential adhesion method (utilizing fibronectin coating) ensured robust SFC attachment within the established principle, while streamlining workflow. This duration effectively isolated high-purity SFCs, as validated by distinct spindle-shaped morphology, positive PRG4 immunofluorescence staining and flow cytometric. Fibronectin coating enhanced adhesion efficiency, enabling specific SFC capture. Both in vitro (Fig1B) and in vivo (Fig1C & Supplementary Figure 1 ) validation by our group confirms the purity of the isolated SFC population. This method was systematically established by Dowthwaite et al. in bovine articular cartilage studies. It involves enzymatic digestion to obtain a full-thickness chondrocyte suspension, which is then seeded onto culture vessels. SFZ chondrocytes rapidly adhere within 2 hours, and non-adherent cells (primarily deep zone chondrocytes) are precisely removed within this time window, successfully yielding a high-purity population of SFZ chondrocytes. These cells highly express progenitor cell markers such as Notch1 and exhibit significant proliferation and chondrogenic potential. Subsequently, McCarthy et al. applied this technique to equine cartilage research, confirming that SFZ chondrocytes isolated via adhesion demonstrate superior expansion efficiency and chondrogenic capacity compared to BMSCs, highlighting its effectiveness in large animal models. The theoretical basis of this method can be traced back to Jones & Watt’s elucidation of the β1-integrin-mediated rapid adhesion mechanism in human epidermal progenitor cells, while Yasuhara et al. further effectively extracted mouse SFZ chondrocytes using this method.

[1] Dowthwaite GP, Bishop JC, Redman SN, Khan IM, Rooney P, Evans DJ, Haughton L, Bayram Z, Boyer S, Thomson B, Wolfe MS, Archer CW. The surface of articular cartilage contains a progenitor cell population. J Cell Sci. 2004 Feb 29;117(Pt 6):889-97. doi: 10.1242/jcs.00912.

[2] McCarthy HE, Bara JJ, Brakspear K, Singhrao SK, Archer CW. The comparison of equine articular cartilage progenitor cells and bone marrow-derived stromal cells as potential cell sources for cartilage repair in the horse. Vet J. 2012 Jun;192(3):345-51. doi: 10.1016/j.tvjl.2011.08.036.

[3] Jones PH, Watt FM. Separation of human epidermal stem cells from transit amplifying cells on the basis of differences in integrin function and expression. Cell. 1993 May 21;73(4):713-24. doi: 10.1016/0092-8674.

[4] Yasuhara R, Ohta Y, Yuasa T, Kondo N, Hoang T, Addya S, Fortina P, Pacifici M, Iwamoto M, Enomoto-Iwamoto M. Roles of β-catenin signaling in phenotypic expression and proliferation of articular cartilage superficial zone cells. Lab Invest. 2011 Dec;91(12):1739-52. doi: 10.1038/labinvest.2011.144.

5.IL-1 Apoptosis Timing: Short-term IL-1 (16h) shows no apoptosis (RTCA, flow cytometry, caspase-3 unchanged), but 72h suppresses proliferation—this time-dependence is well-demonstrated. How does this inform early OA intervention windows?

Answer-5：Our data suggest that IL-1β induces a staged response in SFC. At the early time point, SFC remain viable and apoptosis is not significantly increased, whereas prolonged exposure suppresses proliferative activity. Combined with the early reduction in stemness markers and upregulation of MMP3, these findings suggest that, in early OA, functional impairment of SFC may precede overt cell death. This may indicate a potentially reversible intervention window during which suppressing inflammatory signaling or preserving the progenitor-like phenotype of SFC could delay subsequent degeneration. We have revised the Discussion to clarify this point. Nevertheless, because our conclusions are derived from an in vitro model, the exact clinical intervention window still requires further in vivo validation.

6.Bioinformatics Pathways: GSEA/KEGG shows PI3K-AKT activation and suppressed TNF/IL-17/NF-κB in SFC vs. CC. Could the authors speculate on how this contributes to SFC's anti-apoptotic resistance?

Answer-6: We speculate that the constitutively activated PI3K-AKT pathway in SFC may provide a pro-survival background by supporting cell-survival signaling and anti-apoptotic regulators, whereas the relative suppression of TNF/IL-17/NF-κB signaling may reduce basal inflammatory stress. Together, these molecular features may help explain why SFC show higher Bcl-2 expression, lower caspase activation, and greater resistance to early inflammatory apoptosis than CC. We have added this mechanistic interpretation to the Discussion and clarified that it represents a biologically plausible interpretation based on our transcriptomic and protein data rather than direct pathway-intervention evidence.

7.Stemness Suppression: Early IL-1 markedly reduces CD105/CD34/Sox2 (qPCR Fig 4A). Does this suggest loss of progenitor potential as an early OA event, and was Sox2 protein level validated?

Answer-7: Thank you for this important comment. We agree that the early reduction of CD105, CD34, and SOX2 suggests that suppression of the progenitor-like phenotype may be an early event in OA-related injury of SFC. To address the reviewer’s concern, we further performed Western blot analysis of CD105, CD34, and SOX2 protein expression in control and IL-1β-treated SFC. The protein data showed reduced expression of these stemness-associated markers following early IL-1β intervention, consistent with the qPCR results (new Figure 4B, C). We have therefore revised the Results, Figure 4 legend, and Discussion accordingly. At the same time, we have intentionally used cautious wording throughout the manuscript and describe these findings as early suppression of the progenitor-like phenotype rather than definitive loss of progenitor function, because colony-forming and lineage-differentiation assays were not performed in the present study.

8.Piezo1 Mechanosensitivity: IL-1+CTS-8 upregulates Piezo1 with Ca²⁺ overload (Calcein-AM), F-actin disorganization, and apoptosis. This novel finding warrants emphasis—did inhibitors confirm Piezo1 causality?

Answer-8: Thank you for highlighting this important point. In the present study, we observed that IL-1β combined with CTS-8% was associated with increased Piezo1 expression, intracellular calcium overload, cytoskeletal disorganization, and apoptosis. However, we did not perform pharmacological inhibition or genetic silencing of Piezo1 in this study. Therefore, our current data support a strong association between Piezo1 upregulation and the observed pathological phenotype, but do not establish definitive causality. We have revised the Abstract, Results, and Discussion to use more cautious wording and have added this point as a study limitation. Future experiments using Piezo1 inhibitors or knockdown strategies will be necessary to confirm causality.

9.Immunofluorescence Addition: New IF validation of TNF-α/IL-17/IL-1R1 upregulation (Supp. Fig 3) strengthens RNA-seq/PCR/WB data. Was co-localization with MMP3 assessed to link inflammation to catabolism?

Answer-9: The purpose of Supplementary Figure 3 was to provide immunofluorescence validation of inflammatory activation indicated by RNA-seq, qPCR, and Western blot analyses. Co-localization with MMP3 was not assessed in the current study. Therefore, while our data support concurrent inflammatory activation and MMP3 upregulation following IL-1β exposure, they do not directly demonstrate intracellular co-localization between inflammatory signaling and catabolic responses. We have clarified this point in the revised Discussion and included it as a limitation.

10.MMP3 Biomarker: MMP3 upregulation by IL-1 (qPCR/WB Fig 4F/G) positions it as an early OA marker. Given its collagen-cleaving role, how does this integrate with the cytoskeleton/Piezo1 cascade in Fig 5?

Answer-10: Based on our findings, we propose a two-step pathogenic model. First, IL-1β exposure induces an early inflammatory-catabolic priming state in SFC, characterized by suppression of stemness-associated markers and upregulation of MMP3. Second, under this primed condition, mechanical loading further increases Piezo1 expression, calcium influx, cytoskeletal disorganization, and apoptosis, which may amplify matrix degradation and catabolic signaling. Thus, MMP3 may function as an early catabolic effector within a broader inflammation–mechanotransduction–cytoskeleton cascade. We have expanded the Discussion to better integrate MMP3 into the proposed mechanistic framework while clearly stating that this cascade remains a mechanistic interpretation rather than a fully validated linear pathway.

Attachment Submitted filename: Point-by-point response.docx

# Peer review: Decision Letter 2

**DOI:** 10.1371/journal.pone.0350746.r005

**Authors:** Xindie Zhou

18 May 2026

Molecular Characterization of Superficial Zone Chondrocytes under Pro-Inflammatory and Biomechanical Stress Conditions

PONE-D-25-07335R2

Dear Dr. Wei,

We’re pleased to inform you that your manuscript has been judged scientifically suitable for publication and will be formally accepted for publication once it meets all outstanding technical requirements.

Within one week, you’ll receive an e-mail detailing the required amendments. When these have been addressed, you’ll receive a formal acceptance letter and your manuscript will be scheduled for publication.

An invoice will be generated when your article is formally accepted. Please note, if your institution has a publishing partnership with PLOS and your article meets the relevant criteria, all or part of your publication costs will be covered. Please make sure your user information is up-to-date by logging into Editorial Manager at Editorial Manager® and clicking the ‘Update My Information' link at the top of the page. For questions related to billing, please contact billing support.

If your institution or institutions have a press office, please notify them about your upcoming paper to help maximize its impact. If they’ll be preparing press materials, please inform our press team as soon as possible -- no later than 48 hours after receiving the formal acceptance. Your manuscript will remain under strict press embargo until 2 pm Eastern Time on the date of publication. For more information, please contact onepress@plos.org.

Kind regards,

Xindie Zhou

Academic Editor

PLOS One

Additional Editor Comments (optional):

Reviewers' comments:

Reviewer's Responses to Questions

**Comments to the Author**

1. If the authors have adequately addressed your comments raised in a previous round of review and you feel that this manuscript is now acceptable for publication, you may indicate that here to bypass the “Comments to the Author” section, enter your conflict of interest statement in the “Confidential to Editor” section, and submit your "Accept" recommendation.

Reviewer #2: All comments have been addressed

Reviewer #3: All comments have been addressed

**********

2. Is the manuscript technically sound, and do the data support the conclusions?

The manuscript must describe a technically sound piece of scientific research with data that supports the conclusions. Experiments must have been conducted rigorously, with appropriate controls, replication, and sample sizes. The conclusions must be drawn appropriately based on the data presented.

Reviewer #2: Yes

Reviewer #3: Yes

**********

3. Has the statistical analysis been performed appropriately and rigorously?

Reviewer #2: Yes

Reviewer #3: Yes

**********

4. Have the authors made all data underlying the findings in their manuscript fully available?

The PLOS Data policy requires authors to make all data underlying the findings described in their manuscript fully available without restriction, with rare exception (please refer to the Data Availability Statement in the manuscript PDF file). The data should be provided as part of the manuscript or its supporting information, or deposited to a public repository. For example, in addition to summary statistics, the data points behind means, medians and variance measures should be available. If there are restrictions on publicly sharing data—e.g. participant privacy or use of data from a third party—those must be specified.

Reviewer #2: Yes

Reviewer #3: Yes

**********

5. Is the manuscript presented in an intelligible fashion and written in standard English?

PLOS ONE does not copyedit accepted manuscripts, so the language in submitted articles must be clear, correct, and unambiguous. Any typographical or grammatical errors should be corrected at revision, so please note any specific errors here.

Reviewer #2: Yes

Reviewer #3: Yes

**********

6. Review Comments to the Author

Please use the space provided to explain your answers to the questions above. You may also include additional comments for the author, including concerns about dual publication, research ethics, or publication ethics. (Please upload your review as an attachment if it exceeds 20,000 characters)

Reviewer #2: After careful checking of the revised version, all revision points are fully responded and properly revised. I agree to accept this manuscript for publication.

Reviewer #3: (No Response)

**********

7. PLOS authors have the option to publish the peer review history of their article (what does this mean?). If published, this will include your full peer review and any attached files.

If you choose “no”, your identity will remain anonymous but your review may still be made public.

**Do you want your identity to be public for this peer review?**  For information about this choice, including consent withdrawal, please see our Privacy Policy.

Reviewer #2: No

Reviewer #3: No

**********

# Editor report: Acceptance letter

**DOI:** 10.1371/journal.pone.0350746.r006

**Authors:** Xindie Zhou

PONE-D-25-07335R2

PLOS One

Dear Dr. Wei,

I'm pleased to inform you that your manuscript has been deemed suitable for publication in PLOS One. Congratulations! Your manuscript is now being handed over to our production team.

At this stage, our production department will prepare your paper for publication. This includes ensuring the following:

* All references, tables, and figures are properly cited

* All relevant supporting information is included in the manuscript submission,

* There are no issues that prevent the paper from being properly typeset

You will receive further instructions from the production team, including instructions on how to review your proof when it is ready. Please keep in mind that we are working through a large volume of accepted articles, so please give us a few days to review your paper and let you know the next and final steps.

Lastly, if your institution or institutions have a press office, please let them know about your upcoming paper now to help maximize its impact. If they'll be preparing press materials, please inform our press team within the next 48 hours. Your manuscript will remain under strict press embargo until 2 pm Eastern Time on the date of publication. For more information, please contact onepress@plos.org.

You will receive an invoice from PLOS for your publication fee after your manuscript has reached the completed accept phase. If you receive an email requesting payment before acceptance or for any other service, this may be a phishing scheme. Learn how to identify phishing emails and protect your accounts at https://explore.plos.org/phishing.

If we can help with anything else, please email us at customercare@plos.org.

Thank you for submitting your work to PLOS ONE and supporting open access.

Kind regards,

PLOS ONE Editorial Office Staff

on behalf of

Dr. Xindie Zhou

Academic Editor

PLOS One
